# Inflammation- and resolution-programmed myeloid circuits govern therapeutic resistance in epithelial and mesenchymal triple-negative breast cancer

**DOI:** 10.1172/JCI198815

**Published:** 2026-02-17

**Authors:** Liqun Yu, Charlotte Rivas, Fengshuo Liu, Yichao Shen, Ling Wu, Zhan Xu, Yunfeng Ding, Xiaoxin Hao, Weijie Zhang, Hilda L. Chan, Jun Liu, Bo Wei, Yang Gao, Luis Becerra-Dominguez, Yi-Hsuan Wu, Siyue Wang, Tobie D. Lee, Xuan Li, Xiang Chen, David G. Edwards, Xiang H.-F. Zhang

**Affiliations:** 1 Lester and Sue Smith Breast Center,; 2 Graduate Program in Cancer and Cell Biology,; 3 Graduate Program in Integrative Molecular and Biomedical Sciences,; 4 Medical Scientist Training Program, and; 5 Graduate Program in Immunology and Microbiology, Baylor College of Medicine, Houston, Texas, USA.; 6 Metabolomics Core Facility, Department of Bioinformatics and Computational Biology, The University of Texas MD Anderson Cancer Center, Houston, Texas, USA.; 7 Dan L. Duncan Cancer Center,; 8 Quantitative and Computational Biosciences Graduate Program,; 9 Department of Molecular and Cellular Biology, and; 10 McNair Medical Institute, Baylor College of Medicine, Houston, Texas, USA.

**Keywords:** Immunology, Oncology, Breast cancer, Cancer immunotherapy, Macrophages

## Abstract

Single-cell analysis of human triple-negative breast cancer revealed heterogeneous macrophage populations with opposing phenotypes — proinflammatory and proresolution of inflammation. Paradoxically, both subsets accumulated in therapy-refractory residual tumors but showed inverse correlations across patients, suggesting mutually exclusive resistance mechanisms. Inflammatory macrophages localized preferentially to epithelial-like tumors, whereas proresolution macrophages were enriched in mesenchymal-like tumors. Mouse models faithfully recapitulated these patterns. After chemoimmunotherapy, mesenchymal-like tumors expanded proresolution macrophages through phagocytosis/efferocytosis, ω-3 fatty acid uptake, and resolvin production. Macrophage-secreted C1q emerged as a principal antagonist of T cell function by targeting mitochondria and inducing metabolic dysfunction. By contrast, epithelial-like tumors accumulated inflammatory macrophages and neutrophils that produced prostaglandins via ω-6 fatty acid pathways. Knocking down ELOVL5 — an elongase involved in ω-3 and ω-6 metabolism — mitigated both neutrophil- and macrophage-mediated immunosuppression. These distinct axes, driven by dysregulated inflammation and resolution programs, converged to undermine therapy-induced immunosurveillance; however, targeting their shared upstream regulators may overcome these resistance mechanisms.

## Abstract 2

Single-cell analysis identified two macrophage subsets in triple-negative breast cancer—pro-inflammatory in epithelial tumors, pro-resolution in mesenchymal tumors—each driving therapy resistance converging on Elongase-5 metabolism.

## Graphical abstract

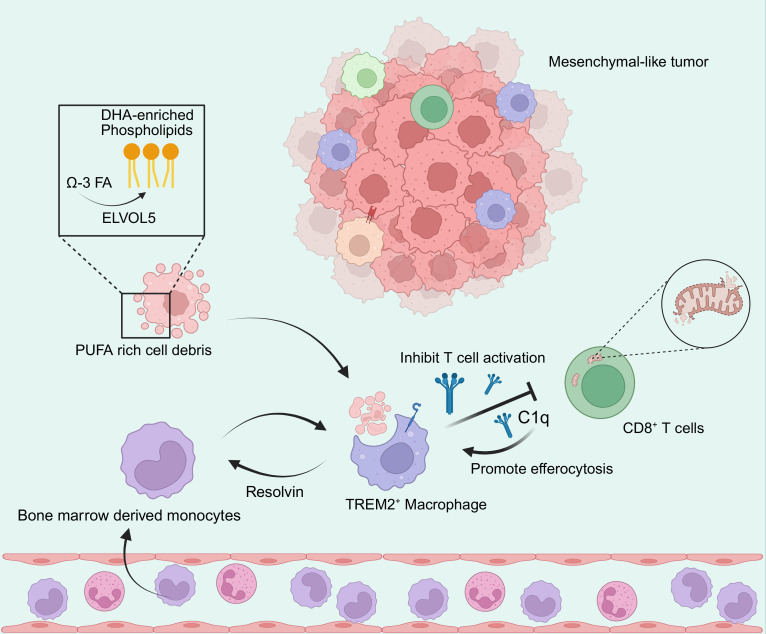

## Introduction

Tumors have been depicted as wounds that never heal (1). Wound healing is an intricate process characterized by an initial inflammatory response, subsequent resolution of inflammation, and eventual tissue remodeling and restoration (2). Within this complex cascade, inflammation and tissue remodeling are increasingly recognized as key determinants of tumor development and therapeutic response (1, 3). The inflammatory response begins with production of soluble mediators, including prostaglandins, CXCL1/2, and IL-1α, orchestrating the recruitment of circulating leukocytes, particularly neutrophils (4). Cancer-associated inflammation is often triggered by growth factors, cytokines, and chemokines resulting from various stresses driven by oncogenic pathways (5, 6). Specifically, damage-associated molecular patterns (DAMPs), produced by cancer cells due to rapid proliferation, apoptosis, and a nutrient-deprived microenvironment, contribute to this inflammatory process (7). Chronic inflammation reciprocally promotes tumor progression by inducing DNA damage and dampening antitumor immunity (8). Additionally, the tissue remodeling phase of wound healing promotes tumor progression, primarily owing to the immunosuppressive milieu and concomitant angiogenesis (9). In normal wound healing, inflammation needs to be resolved before the onset of tissue remodeling. The resolution of inflammation is a highly coordinated process, involving neutrophil apoptosis, macrophage polarization toward a resolution phenotype with enhanced efferocytosis, and increased production of antiinflammatory mediators such as resolvins (10–12). Compared with that of inflammation and tissue remodeling, the contribution of inflammation resolution to cancer progression remains poorly understood.

Macrophages are professional phagocytic cells specialized in detecting, engulfing, and eliminating pathogens and apoptotic cells (7). During inflammation resolution, macrophage-mediated efferocytosis of apoptotic neutrophils drives macrophage polarization into a proresolution status, facilitating the termination of inflammation (12, 13). Macrophages originate either from embryonic precursors that reside in tissues during prenatal development or from bone marrow–derived monocytes that migrate to tissues via a CCR2-dependent mechanism (14). Growth factors like VEGFA and macrophage colony-stimulating factor (M-CSF) also influence monocyte recruitment and differentiation (15, 16). Tumor-associated macrophages (TAMs) exhibit remarkable plasticity, exerting both supportive and inhibitory effects on cancer progression. TAMs can eliminate tumor cells through antibody-dependent phagocytosis (17) and facilitate antitumor therapies by promoting adaptive immune responses (18). Nevertheless, TAMs are primarily recognized for their suppressive functions within tumors, inhibiting cytotoxic T cell activity through various mechanisms. These include producing inhibitory cytokines like IL-10 and TGF-β (19, 20), engagement of immune checkpoints via PD-L1 and B7-H4 expression (21, 22), and consumption of T cell–essential substrates such as L-arginine (23). Additionally, TAMs indirectly influence cytotoxic T cell functions by recruiting immunosuppressive cells like regulatory T cells (24); inhibiting stimulatory immune cells such as dendritic cells and natural killer cells (25); and disrupting vascular structures and remodeling the tumor microenvironment (TME) to induce immune exclusion (26, 27).

The TME evolves as tumor progresses and varies from patient to patient (1, 2). This dynamic and heterogeneous nature has been increasingly appreciated. For instance, several tumor ecotypes have been defined by integrating single-cell and spatial omics data across various cancer types (28). Each ecotype represents a combination of cell types and cell states, characterizing the TME in a subset of patients. Our group previously identified macrophage-enriched and neutrophil-enriched subtypes of TME in preclinical models and clinical specimens of triple-negative breast cancer (TNBC) (29), which reflect divergent tumor ecosystems. Herein, in the macrophage-enriched subtype, we identified a subset of proresolution TAMs as primary mediators of resistance to chemoimmunotherapies, unexpectedly implicating inflammation resolution in suppression of therapy-induced antitumor immunity in specific TMEs.

This study, together with our previous study elucidating a resistance mechanism involving neutrophils and inflammation mediators in neutrophil-enriched TNBC (30), provides evidence that the heterogenous and dynamic TME in different tumors may hijack distinct stages of wound healing mechanisms to evade therapies, thus deepening Dvorak’s concept that “tumor is a wound that never heals” (31).

## Results

### Chemoimmunotherapy-refractory TNBC is characterized by a mutually exclusive enrichment of inflammatory and proresolution macrophages.

**Figure 1:**
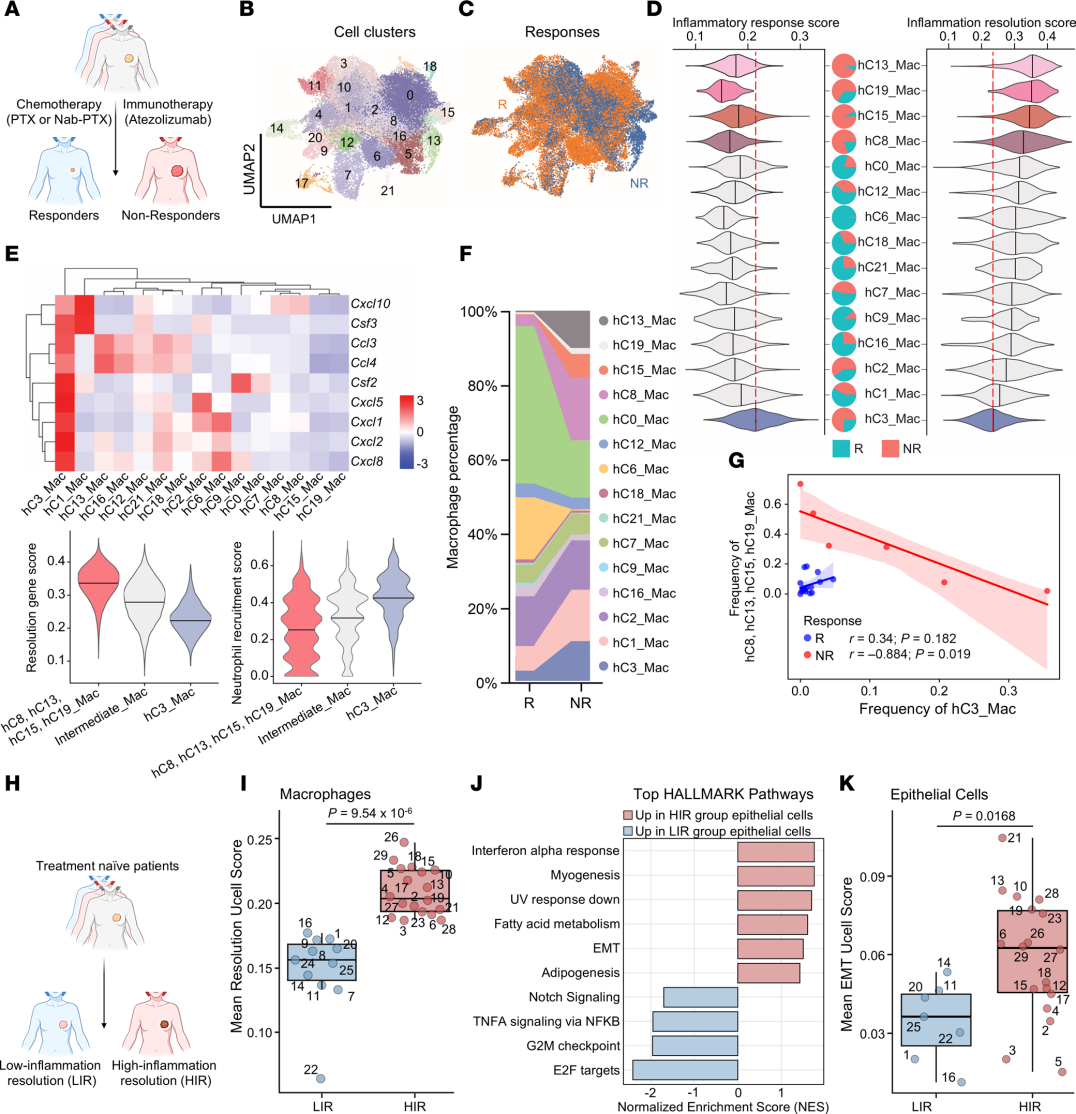
Distinct immune microenvironment in human TNBC. (**A**) Schematic of the patient cohort treated with chemotherapy and immunotherapy, and study design for subsequent analysis. (**B**) Uniform manifold approximation and projection (UMAP) plot of myeloid cell clusters. (**C**) UMAP visualization of myeloid cell distribution between responder and nonresponders. (**D**) Violin plots illustrating AUCell scores for the Hallmark_Inflammatory_Response gene set and the inflammation-resolution gene signature across patient-derived macrophage subclusters. Dashed lines denote the corresponding scores for subcluster hC3_Mac. Pie charts indicate the proportions of responder versus nonresponder cells within each macrophage subcluster. (**E**) Heatmap depicting average expression levels of neutrophil recruitment genes across patient macrophage populations. Violin plots illustrate the distribution of inflammation resolution gene signature scores (lower left) and neutrophil recruitment signature scores (lower right), with horizontal bars representing the mean values. (**F**) Composition analysis of macrophage subsets between responders and nonresponders. (**G**) Pearson correlation analysis of per-patient cell frequencies between human cluster 3 macrophages and cluster 8, 13, 15, and 19 macrophages in responder and nonresponder groups. Shaded areas indicate the 95% confidence interval. Statistical significance was determined using 2-tailed tests. (**H**) Schematic of the treatment-naive patient cohort and study design. (**I**) Box plot of macrophage resolution scores in patients with TNBC, stratified into low- and high-inflammation resolution groups. Each point represents the mean resolution score for macrophages from an individual patient. (**J**) Top Hallmark pathways enriched in epithelial cells of patients with TNBC with low- versus high-inflammation resolution scores. (**K**) Box plot of epithelial-mesenchymal transition (EMT) scores in patients with TNBC. Each point represents the mean EMT score for epithelial cells from an individual patient. (**I** and **K**) A Wilcoxon rank-sum test was used to assess statistical significance between the two groups.

To delineate the dynamics of the tumor immune microenvironment in patients treated with chemotherapy in combination with immune checkpoint blockade (designated hereafter as chemoimmunotherapy), we analyzed single-cell RNA sequencing (scRNA-seq) data from 44 patients with TNBC treated with paclitaxel (PTX, a chemotherapy drug) alone or in combination with atezolizumab (an anti–PD-L1 antibody) (32). Patients were stratified into responders and nonresponders according to their pathological complete response status (Figure 1A). Major myeloid cell populations were identified and subclustered based on distinct gene expression profiles (Supplemental Figure 1A and Figure 1B; supplemental material available online with this article; https://doi.org/10.1172/JCI198815DS1). Comparative analyses between responders and nonresponders revealed notable differences primarily within macrophage subsets (Figure 1C). Interestingly, among 15 human macrophage clusters, those with the highest inflammation-resolution AUCell scores (hC8, hC13, hC15, and hC19, with “h” denoting “human,” to be distinguished from murine data shown later) were significantly enriched in nonresponder tumors (Figure 1D and Supplemental Figure 1B). Conversely, cluster hC3 — marked by an inflammatory gene program opposite to the resolution phenotype — was also enriched in nonresponders (Figure 1D and Supplemental Figure 1, C and D). These macrophages demonstrated elevated expression of neutrophil recruitment–associated genes (Figure 1E). Despite the notoriously high dropout rate of neutrophils in scRNA-seq data due to technical caveats (33, 34), the abundance of hC3 macrophages suggests a neutrophil-enriched inflammatory TME.

Both inflammation-resolution macrophages (hC8, hC13, hC15, hC19) and inflammatory macrophages (hC3) were enriched in nonresponsive tumors (Figure 1F and Supplemental Figure 1E) yet segregated by patients. Correlation analysis revealed a significant negative association between these two programs among nonresponders, indicating mutual exclusivity. In contrast, no such relationship was observed in responders (Figure 1G).

Collectively, these findings suggest that both inflammatory and antiinflammatory macrophages can underlie resistance to chemoimmunotherapy: the former through recruiting and reprogramming neutrophils as we previously elucidated (30), and the latter by exploiting inflammation-resolution pathways.

### Epithelial-mesenchymal transition status correlates with tumor-infiltrating macrophage polarization.

To extend our analysis to additional clinical samples and investigate tumor-intrinsic characteristics potentially underlying macrophage phenotypic divergence, we examined an integrated scRNA-seq dataset (35) containing cancer cell data of the same patients. This dataset consists of 29 treatment-naive patients with primary TNBC. Patients were classified into low inflammation-resolution (LIR) and high inflammation-resolution (HIR) groups based on the macrophage inflammation-resolution gene expression (Figure 1, H and I, and Supplemental Figure 1, F–H).

To assess whether these distinct macrophage phenotypes correlate with intrinsic properties of cancer cells, we conducted gene set enrichment analysis (GSEA) on epithelial cells. Cancer cells from the LIR group demonstrated enrichment in Notch signaling, E2F targets, G2M checkpoint, and TNF-α signaling pathways, suggesting accelerated tumor growth (Figure 1J). Interestingly, epithelial cells in the HIR group exhibited elevated expression in the epithelial-mesenchymal transition (EMT) pathway. Further analysis confirmed higher EMT scores in cancer cells derived from HIR tumors (Figure 1K and Supplemental Figure 1I).

Collectively, these findings indicate that the inflammation-resolution program of macrophages correlates with elevated EMT status in cancer cells.

### EMT status of murine TNBC models correlates with myeloid cell profiles.

To better understand how inflammation-resolution programmed macrophages drive therapy resistance, we established syngeneic murine TNBC models that recapitulate key tumor and immune characteristics observed in human TNBC and analyzed their transcriptomic profiles. Cell lines exhibiting elevated expression of mesenchymal genes (Supplemental Figure 2, A and B) demonstrated reduced expression of Hallmark Inflammation Response gene signature (Supplemental Figure 2, C and D). In contrast, cell lines retaining epithelial features showed increased expression of inflammation-associated genes, suggesting that EMT-associated immune shifts are conserved in murine TNBC.

**Figure 2:**
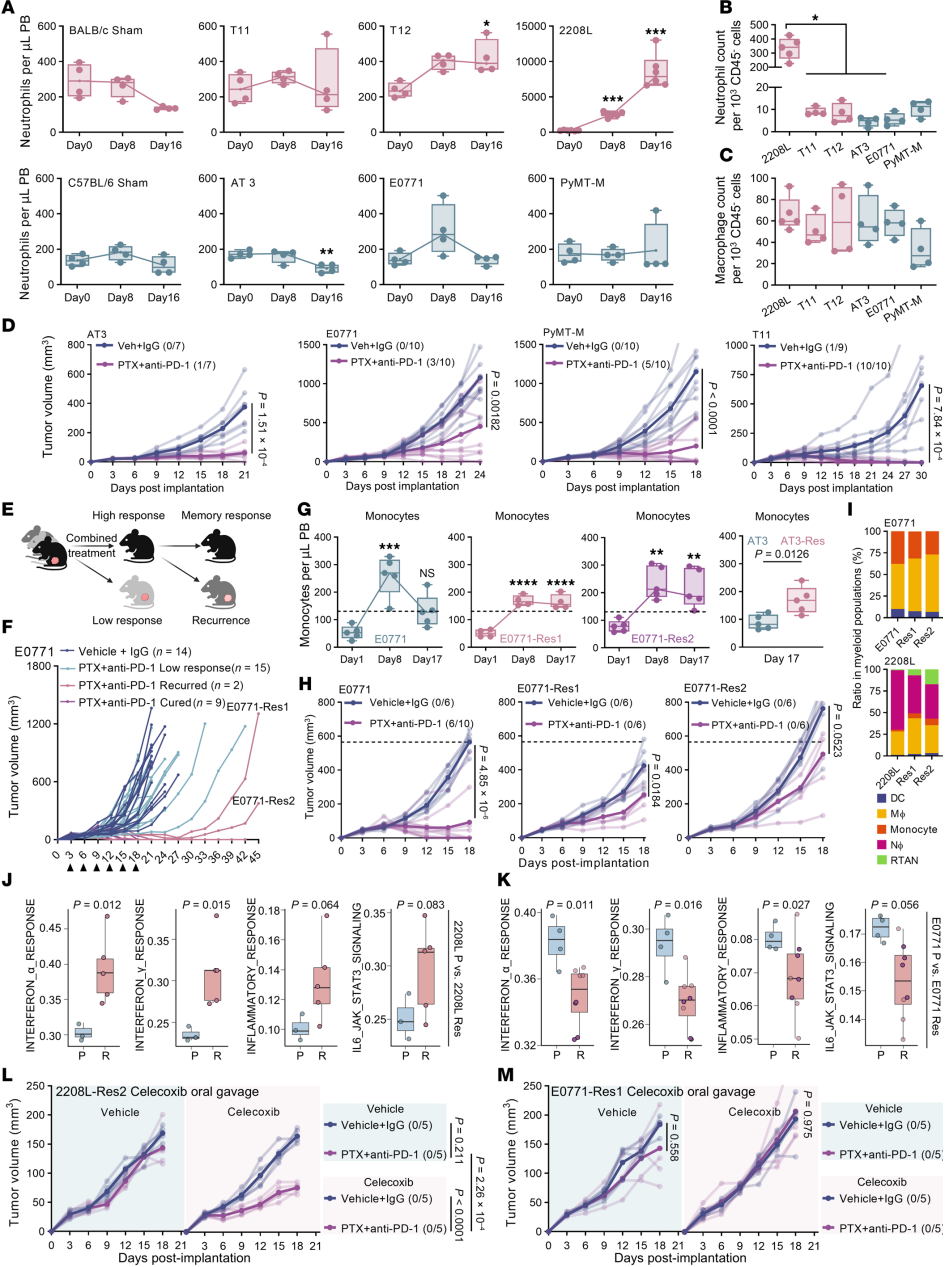
Dichotomous immune microenvironment in murine TNBC models. (**A**) Flow cytometry analysis of peripheral blood (PB) neutrophils from BALB/c mice (pink) with sham (surgery without tumor implantation), T11, T12, and 2208L tumors and C57BL/6 mice (blue) with sham, AT3, E0771, and PyMT-M tumors. (**B** and **C**) Neutrophil (**B**) and macrophage (**C**) infiltration in the indicated tumors at day 20 after implantation. (**D**) Growth of AT3, E0771, PyMT-M, and T11 tumors under vehicle or paclitaxel (PTX) plus anti–PD-1 antibody. Numbers indicate cured/total mice per group. Dark lines represent group averages; light lines represent individual tumor size. (**E**) Schematic of the experimental design: following combined treatment, mice with tumor eradication were monitored for recurrence. Recurrence-free mice were rechallenged to assess immune memory. Relapsed tumors were excised to generate resistant cell lines. (**F**) Growth of E0771 tumors under vehicle or combined treatment. (**G**) Analysis of PB monocytes from mice with E0771, E0771-Res1, E0771-Res2 tumors and from mice with AT3 and AT3-Res tumors. (**H**) Growth of E0771, E0771-Res1, and E0771-Res2 tumors under vehicle or PTX plus anti–PD-1 antibody. (**I**) Average myeloid cell infiltrates in 2208L and E0771 tumors and their resistant derivatives (*n* = 3) determined by single-cell RNA-seq. (**J** and **K**) Relative expression of selected Hallmark gene sets in 2208L and E0771 parental tumors and resistant derivatives by bulk RNA-seq. Points represent individual samples, which are color-coded by group. (**J**) 2208L parental (P, *n* = 3), 2208L-resistant tumors (R, *n* = 5). (**K**) E0771 parental (P, *n* = 4), E0771-Res1 (purple, *n* = 4), and E0771-Res2 (pink, *n* = 4). (**L** and **M**) Tumor growth of 2208L-Res2 (**L**) and E0771-Res1 (**M**) tumors receiving vehicle or 100 mg/kg celecoxib daily and treated with vehicle or combined treatment. (**A**–**C**, **G**, **L**, and **M**) Statistical significance was determined using 1-way ANOVA followed by Tukey’s test. (**D**, **G**, and **H**) Significance was calculated using unpaired 2-tailed Student’s *t* test. *P < 0.05, **P < 0.005, ***P < 0.001, ****P < 0.0001.

When implanted into mice, epithelial-like tumors (2208L and EMT6) induced a pronounced systemic neutrophilia (Figure 2A and Supplemental Figure 2E). By contrast, mesenchymal-like tumors (T11, T12, AT3, E0771, PyMT-M) left neutrophil counts largely unchanged (Figure 2A); however, only the T12 model uniquely drove a marked expansion of circulating monocytes (Supplemental Figure 2F).

Within the TEM, epithelial-like 2208L tumors exhibited robust neutrophil infiltration (Figure 2B), while mesenchymal-like tumors maintained low neutrophil frequencies and comparable macrophage levels (Figure 2C). Moreover, the few neutrophils present in mesenchymal-like tumors were primarily localized to apoptotic regions, whereas neutrophils in 2208L tumors were abundantly and uniformly distributed throughout the tumor (Supplemental Figure 2G). scRNA-seq analysis of tumor-infiltrating myeloid cells suggested that macrophages within mesenchymal-like tumors exhibited enhanced hemophagocytic activity, consistent with an inflammation-resolution phenotype (Supplemental Figure 2H).

Combined chemoimmunotherapy produced mixed outcomes: while most mesenchymal-like tumors regressed (Figure 2D), the T12 model exhibited de novo resistance, accompanied by a systemic surge in circulating monocytes (Supplemental Figure 2I).

### Mesenchymal-like tumors acquire therapeutic resistance through exacerbating inflammation-resolution.

Following 6 cycles of combined therapy, tumor-bearing mice exhibited heterogeneous responses (Figure 2, E and F). Mice that achieved complete tumor eradication developed immunological memory, providing protection against subsequent rechallenge (Supplemental Figure 2J). However, a subset of tumors evaded immune surveillance and relapsed after therapy cessation (Figure 2F). To investigate this further, we excised relapsed tumors and established resistant cell lines: E0771-Res1, E0771-Res2, and AT3-Res.

Resistant derivatives of E0771 and AT3 displayed sustained increases of monocytes in the peripheral blood (Figure 2G and Supplemental Figure 2K), while neutrophil levels remained unchanged (Supplemental Figure 2, L and M). In contrast, monocyte levels were only transiently elevated in mice bearing parental E0771 tumors and exhibited a modest late-stage increase in mice bearing parental AT3 tumors. Notably, these resistant derivatives were refractory to both monotherapies and the combined regimen (Figure 2H and Supplemental Figure 2, N–P).

Previously, we reported that resistant epithelial-like 2208L tumors evade immune surveillance by reprogramming neutrophils into an immunosuppressive phenotype (30). By contrast, mesenchymal-like resistant E0771 tumors remained neutrophil-scarce, and macrophages constituted a larger fraction of the myeloid compartment compared with parental tumors (Figure 2I). Strikingly, transcriptomic comparisons between resistant derivatives and their parental counterparts revealed divergent pathway alterations: 2208L-resistant tumors demonstrated elevated expression of proinflammatory pathways, including IFN-α, IFN-γ, and IL-6 signaling (Figure 2J), whereas these same pathways were downregulated in E0771-resistant tumors (Figure 2K).

To functionally distinguish the resistance mechanisms between epithelial- and mesenchymal-like cancers, mice bearing resistant tumors were orally administered 100 mg/kg celecoxib, a nonsteroidal antiinflammatory drug. Celecoxib improved the efficacy of combined treatment in 2208L-resistant tumors (Figure 2L) but had minimal effect on E0771-resistant tumors (Figure 2M).

Collectively, these findings reveal 2 distinct TMEs driven by tumor-intrinsic states. Epithelial-like tumors maintain a proinflammatory milieu dominated by neutrophils, whereas mesenchymal-like tumors recruit inflammation-resolution macrophages, creating an antiinflammatory microenvironment. Therapy resistance further polarizes these divergent TMEs.

### Acquisition of therapeutic resistance in mesenchymal-like tumors is accompanied by expansion of TREM2^+^ macrophages.

**Figure 3:**
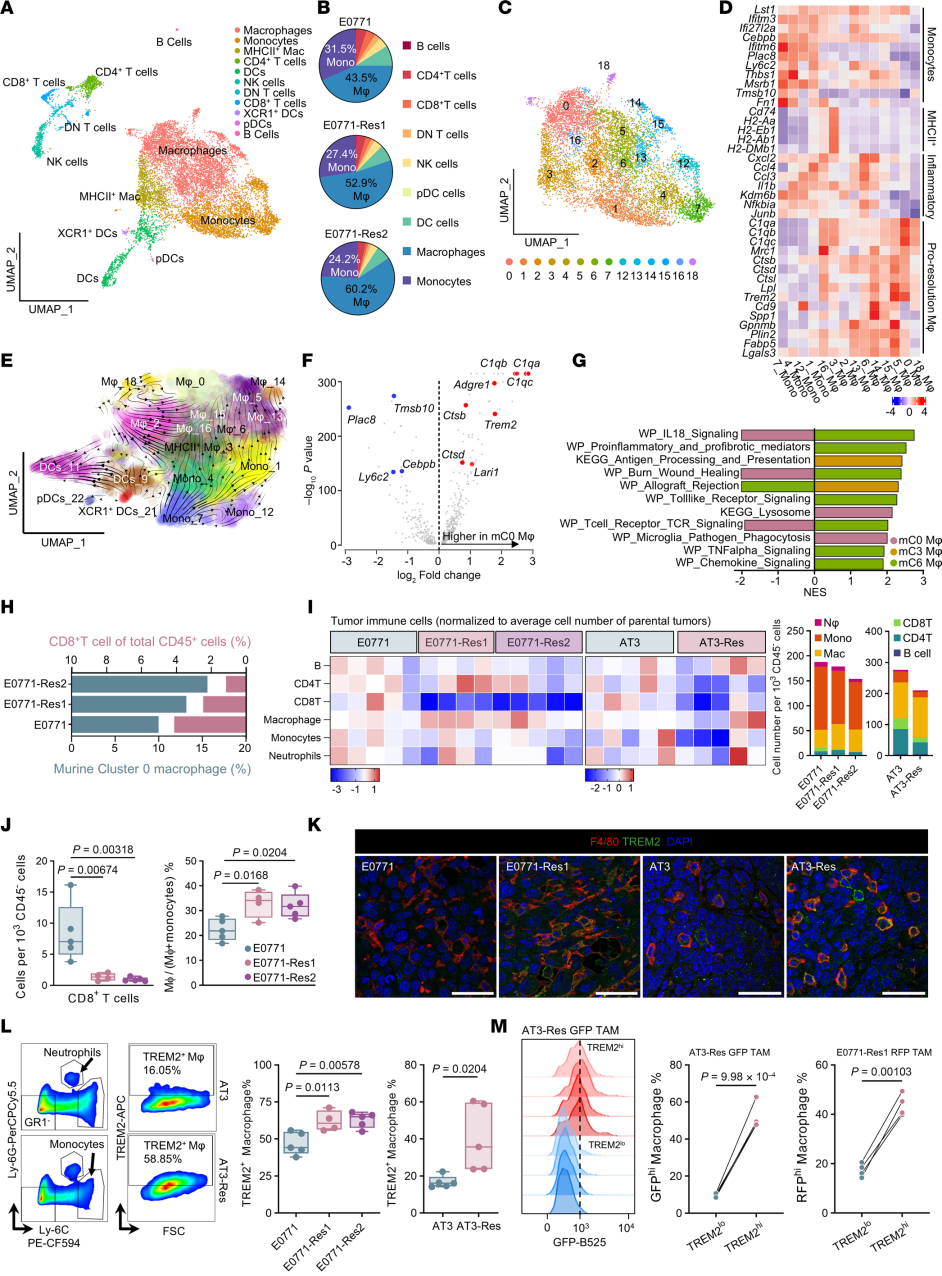
Resistant mesenchymal-like tumors promote tumor-associated macrophage differentiation. (**A**) UMAP visualization of immune cells from merged tumor samples. (**B**) Pie charts illustrate immune cell composition in parental E0771 and resistant tumors. (**C**) UMAP of monocyte and macrophage clusters from merged tumor samples. (**D**) Heatmap of average expression of monocyte, MHCII^+^, inflammatory, and inflammation-resolution macrophage signatures across macrophage/monocyte subclusters. (**E**) Predicted cell-state transitions overlaid on the myeloid UMAP. (**F**) Volcano plot illustrates differential gene expression between murine cluster 0 macrophages and other clusters. *P* values were determined by Wilcoxon’s rank-sum test. (**G**) GSEA for macrophage-specific signature genes from murine clusters 0, 3, and 6. Normalized enrichment score (NES)value indicates enrichment score of specified cluster against all other clusters combined. (**H**) Ratio of murine cluster 0 macrophages and CD8^+^ T cells within CD45^+^ immune cells. (**I**) Flow cytometry of immune infiltrates in E0771, AT3, and their resistant derivatives at endpoint. (Left) Log_2_ fold change relative to the vehicle-treated parental tumors; each square represents 1 cell type per tumor. (Right) Average immune cell number per 1,000 CD45^–^ cells (*n* = 5). (**J**) Flow cytometry of CD8^+^ T cells (left) and macrophage/(monocyte+macrophage) ratio (right) in E0771, E0771-Res1, and E0771-Res2 tumors. Significance was determined using 1-way ANOVA followed by Tukey’s test. (**K**) Representative immunofluorescence of F4/80, TREM2, and DAPI in parental and resistant tumors. Scale bar: 40 μm. (**L**) Representative flow plots (left) and quantification of TREM2^+^ macrophages in E0771, AT3, and resistant derivatives. Significance for the middle plot was determined using 1-way ANOVA followed by Tukey’s test. Significance for the right plot was calculated using unpaired 2-tailed Student’s *t* test. (**M**) Representative flow plots (left) and quantification of fluorescence intensity in TREM2^lo^ and TREM2^hi^ tumor-associated macrophages (TAMs) in AT3-Res GFP and E0771-Res1 RFP tumors. Significance was calculated using paired 2-tailed Student’s *t* test (*n* = 4).

To further characterize the acquired resistance in inflammation-resolution macrophage-enriched TME, we conducted scRNA-seq on parental E0771 tumors and 2 resistant variants. Eighteen days after implantation, tumors from 3 animals per model were excised, and CD45^+^ immune cells were isolated and sequenced. In total, 11,931 cells were analyzed (Figure 3A), 23 cell clusters were resolved, and 11 cell types were identified based on canonical marker genes (Supplemental Figure 3A). Myeloid cells, primarily monocytes and macrophages, represented over 80% of the immune cells in tumors (Figure 3B). Notably, resistant tumors exhibited increased myeloid cell proportions and a higher macrophage-to-monocyte ratio, indicating enhanced monocyte recruitment and differentiation into macrophages within the resistant TME.

To further dissect monocyte/macrophage heterogeneity, we utilized existing reference sequencing data to annotate these cells (36), identifying 4 distinct groups (Supplemental Figure 3B and Figure 3, C and D). These groups were distinguished by their expression of canonical markers: monocytes were represented by cluster 7 (mC7, with “m” designating “murine”) (*Fn1*, *Ly6c2*, *Ifitm6*), MHCII^+^ were macrophages represented by cluster 3 (mC3), (*Cd74*, *H2-Eb1*, *H2-Ab1*), inflammatory macrophages were represented by cluster 6 (mC6) (*Cxcl2*, *Ccl3*, *Ccl4*), and inflammation-resolution macrophages were represented by clusters 0 and 5 (mC0 and mC5) (*Trem2*, *Mrc1*, *Ctsd*). Feature plots validated these classifications. Furthermore, *Adgre1*, encoding the macrophage marker F4/80 (Supplemental Figure 3C), and complement component 1q genes (*C1qa*, *C1qb*, and *C1qc*) were also enriched in cluster 0 macrophages (Figure 3D).

Cluster distribution shifted from predominantly MHCII^+^ macrophages (mC3) (25.9%) in parental E0771 tumors toward TREM2^+^C1q^+^ proresolution macrophages (mC0) (24.9% and 25.9%) in resistant variants (Supplemental Figure 3D). This shift aligned with bulk RNA-seq results indicating reduced inflammatory activity in resistant tumors (Figure 2K). Interestingly, TREM2^+^ macrophages were diminished in 2208L-resistant tumors, whereas inflammatory macrophages increased (Supplemental Figure 3, E and F), underscoring distinct immune evasion strategies between epithelial- and mesenchymal-like tumor models.

Using *Dynamo*, a trajectory inference tool, we explored the cell fate transitions within the myeloid population. The analysis highlighted a gradual progression starting with monocytes (mC7) that transitioned to MHCII^+^ macrophages (mC3) and inflammatory macrophages (mC6) and, ultimately, evolved into TREM2^+^ C1q^+^ macrophages (mC0) (Figure 3E). These macrophages also decreased monocyte markers compared with other macrophages (Figure 3F). To further delineate the functional attributes of these macrophage populations, we conducted GSEA using cluster-specific gene signatures. mC3 macrophages appeared to enrich antigen processing and presentation pathways, and mC6 macrophages were characterized by activated inflammation-related signaling, including TNF-α, chemokine, and T cell receptor pathways (Figure 3G). In contrast, mC0 macrophages exhibited decreased IL-18 and TCR signaling but enhanced phagocytosis and lysosomal pathways. Notably, resistant tumors exhibited increased TREM2^+^C1q^+^ macrophages and reduced CD8^+^ T cells compared with parental tumors (Figure 3H), consistent with a more immunosuppressive TEM.

We next assessed the expression of genes that defined the mC0 cluster (designated hereafter as the mC0 macrophage signature) in the well-defined M1 and M2 macrophages. Stimulation with M-CSF and IL-4 in vitro effectively upregulated MRC1 and *Arg1* (Supplemental Figure 3, G and H), while M-CSF with IFN-γ and LPS robustly increased MHCII expression and substantially downregulated MRC1 (Supplemental Figure 3G). Notably, M-CSF treatment alone elevated TREM2 expression to an extent similar to that of M2 macrophages and induced a strong expression of the mC0 gene signature (Supplemental Figure 3H). In contrast, macrophages treated with M-CSF and TNF-α displayed reduced expression of all 3 surface markers (Supplemental Figure 3G). Overall, TREM2^+^ macrophages are phenotypically similar to M2 macrophages but are distinguished by higher expression of *C1q* genes and increased secretion of C1q protein (Supplemental Figure 3I).

To validate the scRNA-seq results, we examined tumor-infiltrating immune cells within E0771 and AT3 tumors. In both models, resistant tumors exhibited significant enrichments of TREM2-expressing macrophages, along with notably reduced T cell infiltration (Figure 3, I–L, and Supplemental Figure 3, J–L). Additionally, using fluorescence-labeled tumor cells, we confirmed that TREM2^hi^ macrophages demonstrated an enhanced ability to engulf cancer cell debris, as evidenced by increased fluorescence detection within these macrophages (Figure 3M).

We then investigated whether this resistant phenotype was also present in other mesenchymal models. AT3 and T12 tumors exhibited the highest macrophage-to-monocyte ratios of examined tumor types (Supplemental Figure 3M). In comparison to the other p-53 null model, T11, T12 tumors demonstrated strong recruitment of TREM2^+^ macrophages and significantly reduced CD8^+^ T cells (Supplemental Figure 3, N–Q). Collectively, mice bearing resistant tumors demonstrated increased monocyte recruitment and enhanced differentiation into TREM2^+^ macrophages, which possess potent phagocytic and efferocytosis capabilities.

### Monocyte-derived TAMs are determinants of therapy resistance.

**Figure 4:**
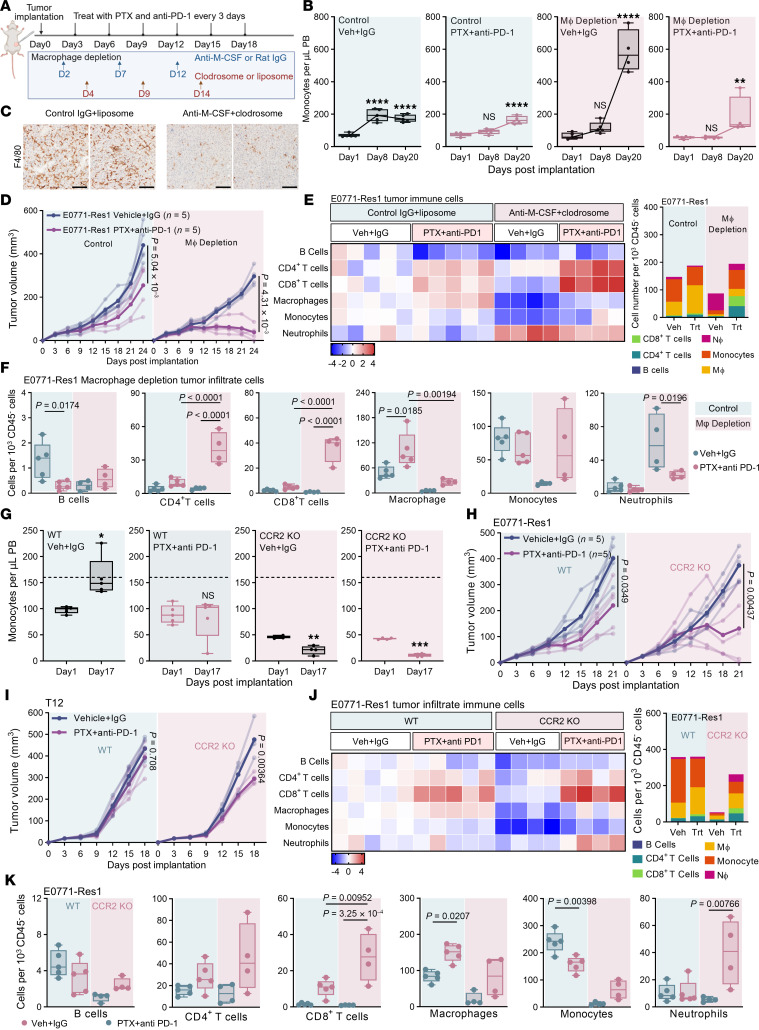
Monocyte-derived tumor-associated macrophages are key mediators of therapy resistance. (**A**) Schematic of the experimental design: Combined treatments started on day 3 after tumor implantation. Anti–M-CSF plus clodrosome or control IgG1 plus liposome were administered as indicated. (**B**) Flow cytometry of PB monocytes in E0771-Res1 tumor-bearing mice under control or combined therapy, with or without macrophage depletion (*n* = 5). (**C**) Representative immunohistochemistry of F4/80 in E0771-Res1 tumors treated with control IgG plus liposome or anti–M-CSF plus clodrosome. Scale bar: 100 μm. (**D**) Tumor growth of E0771-Res1 tumors under vehicle or combined therapy, with or without macrophage depletion (*n* = 5). The values 5.04 × 10^3^ and 4.31 × 10^–4^ represent *P* values for the comparison of tumor volumes between combined paclitaxel and anti–PD-1 treatment and vehicle control at day 24 in control and macrophage-depleted mice, respectively. (**E**) Flow cytometry of immune infiltrates in E0771-Res1 tumors at endpoint. (Left) Log_2_ fold change of major immune cells relative to the vehicle-treated tumor group. (Right) Average immune cell number per 1,000 CD45^–^ cells. (**F**) Quantification of major immune cells per 1,000 CD45^–^ cells in E0771-Res1 tumors under indicated treatments. (**G**) PB monocytes in E0771-Res1 tumor-bearing WT or CCR2-KO mice treated with control or combined treatment. Samples collected on day 1 and 17 after implantation. Significance was calculated using paired 2-tailed Student’s *t* test. (**H** and **I**) Growth of E0771-Res1 (**H**) and T12 tumors (**I**) in WT or CCR2-KO mice treated with vehicle or PTX combined with anti–PD-1 (*n* = 5 mice per group). (**J**) Flow cytometry analysis of the immune infiltrates in E0771-Res1 tumors. (Left) Log_2_ fold change of major immune cells relative to the vehicle-treated tumor group. (Right) Average immune cell number per 1,000 CD45^–^ cells. (**K**) Quantification of major immune cells per 1,000 CD45^–^ cells in E0771-Res1 tumors in WT versus CCR2-KO mice. (**B**, **D**, **F**, **H**, **I**, and **K**) Statistical significance was determined using 1-way ANOVA followed by Tukey’s test. *P < 0.05, **P < 0.005, ***P < 0.001, ****P < 0.0001.

The contribution of TAMs to therapeutic resistance has been extensively documented (37). Our data indicate that a distinctive mC0 TREM2^+^C1q^+^ macrophage subset underlies the acquired resistance observed in macrophage-rich TNBC. To evaluate the causal role of TAMs in this context, we depleted macrophages in mice bearing resistant tumors using an anti–M-CSF monoclonal antibody combined with clodrosome (Figure 4A). While circulating monocyte levels remained stable initially, a significant increase was observed by day 20 (Figure 4B), suggesting enhanced monocyte release into the bloodstream to compensate for macrophage loss in peripheral tissues (Figure 4C). Notably, macrophage depletion markedly improved the efficiency of combined therapy (Figure 4D). In the T12 model with de novo resistance, macrophage depletion and combined therapy also substantially reduced tumor growth (Supplemental Figure 4A). In both models, macrophage depletion resulted in a higher proportional representation of neutrophils and lymphoid (Figure 4E and Supplemental Figure 4, B and C). In E0771-Res1 tumors, the combination therapy and macrophage depletion synergistically enhanced T cell infiltration, yielding a markedly higher proportion of T cells relative to total CD45^–^ cells (Figure 4F). In contrast, macrophage depletion in mice bearing 2208L-resistant tumors exacerbated systemic neutrophil enrichment on day 8 (Supplemental Figure 4D) yet had no effect on treatment outcome or intratumoral neutrophil influx (Supplemental Figure 4, E–H).

Our data indicate that mC0 TREM2^+^C1q^+^ macrophages likely originate from circulating monocytes rather than tissue-resident macrophages. To verify this, we employed a Ms4a3-Cre; Ai14(tdTomato) lineage-tracing system, which selectively labels bone marrow–derived monocytes and macrophages with tdTomato (38) (Supplemental Figure 4I). We demonstrated that tdTomato^+^ bone marrow–derived macrophages (BMDMs) express significantly higher TREM2 expression than tdTomato^–^ tissue-resident macrophages (Supplemental Figure 4J). To further evaluate the functional relevance, we utilized Ccr2^tm1Ifc^ (CCR2-KO) mice (39), characterized by impaired monocyte recruitment (Figure 4G). CCR2 deficiency greatly improved the efficacy of combined therapy, reducing tumor size across all 3 models tested (Figure 4, H and I, and Supplemental Figure 4K). These KO mice also displayed increased infiltration of lymphoid populations relative to CD45^–^ cells (Figure 4J and Supplemental Figure 4L), particularly T cells (Figure 4K and Supplemental Figure 4M). Collectively, these findings suggest that monocyte-derived TREM2^+^ macrophages contribute to immune evasion and play a critical role in therapy resistance in mesenchymal-like tumor models.

### Efferocytosis and resolvin production promote TREM2^+^ macrophage differentiation.

**Figure 5:**
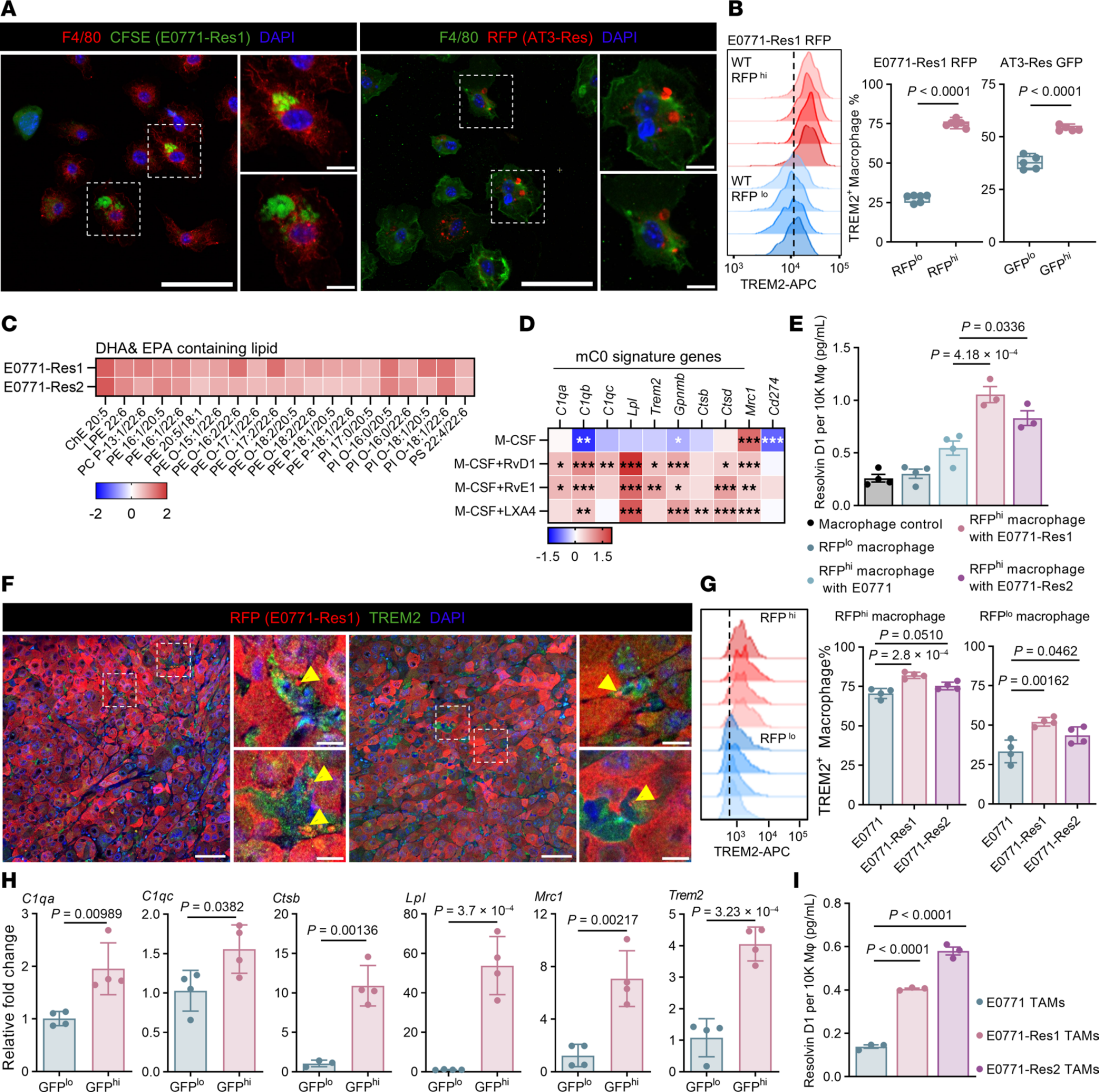
Efferocytosis and resolvin production promote inflammation resolution macrophage differentiation. (**A**) Representative immunofluorescence of F4/80, CFSE (left), RFP (right), and DAPI in macrophages cocultured with paclitaxel-treated (PTX-treated) E0771-Res1 (left) or AT3-Res RFP (right) cells. Scale bar: 50 μm; 10 μm (zoomed-in). (**B**) Flow cytometry (left) and quantification of TREM2 expression in RFP^lo^ versus RFP^hi^ macrophages after coculture with PTX-treated, RFP-labeled E0771-Res1 (middle, *n* = 6), or GFP^lo^ versus GFP^hi^ macrophages after coculture with PTX-treated, GFP-labeled AT3-Res cells (right, *n* = 5). (**C**) Lipidomic comparison of DHA- and EPA-containing lipids in resistant versus parental E0771 cells (*n* = 3). Lipids with absolute log_2_ fold change > 0.5 and *P* < 0.05 were included. (**D**) Relative expression of murine cluster 0 macrophage-specific genes in bone marrow–derived macrophages (BMDMs, *n* = 3) treated with M-CSF (5 or 10 ng/mL) alone or M-CSF (5 ng/mL) plus resolvin D1 (RvD1), resolvin E1 (RvE1), or lipoxin A4 (LXA4, 30 ng/mL). BMDMs treated with M-CSF (5 ng/mL) alone served as baseline control. (**E**) ELISA quantification of RvD1 in BMDMs cocultured with RFP-labeled, PTX-treated E0771 (*n* = 4), E0771-Res1 (*n* = 3), and E0771-Res2 cells (*n* = 3). Macrophages were sorted by RFP signal. Macrophage cultured alone (*n* = 4) served as baseline control. (**F**) Representative immunofluorescence of RFP, TREM2, and DAPI in E0771-Res1 tumors. Arrowheads highlight engulfed RFP debris. Scale bar: 50 μm; 10 μm (zoomed-in). (**G**) Flow cytometry plots (left) and quantification of TREM2 expression in RFP^hi^ (middle) and RFP^lo^ (right) tumor-associated macrophages (TAMs) from RFP-labeled E0771, E0771-Res1, and E0771-Res2 tumors (*n* = 4). (**H**) Relative expression of murine cluster 0 macrophage-specific genes in TAMs from GFP-labeled AT3-Res tumors (*n* = 4). (**I**) ELISA analysis of RvD1 in TAMs from E0771, E0771-Res1, and E0771-Res2 tumors (*n* = 3). (**D**, **E**, **G**, and **I**) Significance was determined using 1-way ANOVA followed by Tukey’s test. (**B** and **H**) Significance was calculated using paired 2-tailed *t* test. *P < 0.05, **P < 0.005, ***P < 0.001.

Given the enrichment of phagocytosis/efferocytosis–related pathways in mC0 macrophages, we investigated whether efferocytosis is crucial for TREM2^+^ macrophage differentiation and their involvement in resistance development. When BMDMs were cocultured with fluorescence-labeled, PTX-pretreated tumor cells, macrophages efficiently internalized the fluorescent debris (Figure 5A). Treatment with the phagocytosis inhibitor cytochalasin D markedly reduced cell debris uptake (Supplemental Figure 5A). Importantly, macrophages with higher fluorescence intensities also exhibited increased surface TREM2 expression and elevated mC0 signature gene expression (Figure 5B and Supplemental Figure 5B). These data suggest a strong association between efferocytosis and inflammation-resolution macrophage differentiation.

TREM2, known for its role in detecting damage-associated lipid patterns, is critical for sensing and clearing lipids linked to cellular damage (40). Lipidomics analysis demonstrated increased triglycerides levels and elevated membrane phospholipids, such as phosphoserine and phosphocholine in E0771-resistant cells as compared with their parental cells (Supplemental Figure 5C). While neutral lipids accumulated in resistant 2208L derivatives and lipid droplet (LD) disruption reversed their resistance (30) (Supplemental Figure 5D), disruption of LDs in E0771-resistant cells by perilipin knockdown (Supplemental Figure 5E) failed to restore sensitivity to therapy despite similar triglyceride enrichment (Supplemental Figure 5F). This again highlights distinct lipid-associated resistance mechanisms between these tumor models.

Further lipid characterization revealed increased unsaturation in phospholipids from E0771-resistant derivatives (Supplemental Figure 5G), indicating enrichment of unsaturated fatty acid within their membranes. Additionally, we observed elevated levels of lipids containing docosahexaenoic acid (DHA), eicosapentaenoic acid (EPA), and arachidonic acid (AA) (Figure 5C and Supplemental Figure 5H). Given the established antiinflammatory roles of DHA and EPA, we evaluated their potential in inducing mC0 macrophage gene signatures. DHA and EPA in combination with M-CSF enhanced most mC0 signature genes compared with M-CSF alone or combined with AA. However, all these treatments minimally induced *C1q* genes that distinguish mC0 macrophages (Supplemental Figure 5I).

Despite no direct effects of DHA, EPA, and AA on TREM2^+^ macrophage differentiation, their metabolites — antiinflammatory mediators such as resolvin D, resolvin E, and lipoxin A4 — significantly upregulated mC0 gene expression (Figure 5D). ELISA analysis revealed that among the 3 aforementioned mediators, only resolvin D1 (RvD1) was detectable in macrophages cocultured with TNBC cells. Macrophages cocultured with unlabeled resistant cells produced more RvD1 compared with those cocultured with parental cells (Supplemental Figure 5J). Additionally, macrophages that phagocytosed E0771 cell debris produced marginally more RvD1 than macrophages that were cultured alone or macrophages that were in coculture but not undergoing phagocytosis (remaining fluorescence-negative) (Figure 5E). Moreover, BMDMs treated with RvD1 in combination with M-CSF produced significantly greater amounts of C1q than those treated with M-CSF alone (Supplemental Figure 5K). Notably, macrophages ingesting debris from resistant cells exhibited significant increases in RvD1 production. TAMs also displayed phagocytic activity within the tumor (Figure 5F), and the TAMs with higher fluorescence signals showed elevated TREM2 expressions and increased mC0 signature expression (Figure 5, G and H). TAMs from resistant tumors expressed higher TREM2 levels in both fluorescence-positive and negative populations (Figure 5G). Furthermore, TAMs in resistant tumors also produced significantly more RvD1 than their parental counterparts (Figure 5I). Together, these data suggest that elevated RvD1 paracrine signaling in the resistant TEM may underlie the observed increase in TREM2^+^ TAMs.

To validate the mechanistic findings in human TNBC models, we cocultured THP-1–derived macrophages with PTX-pretreated, CFSE-labeled human TNBC cells, including the MDA-MB-231 cell line and the PDX-derived WHIM12 line (Supplemental Figure 5L). Macrophages that engulfed tumor cell debris displayed elevated surface TREM2 expression (Supplemental Figure 5M) and increased RvD1 production (Supplemental Figure 5N). Furthermore, macrophages treated with RvD1 secreted significantly higher levels of C1q into the culture medium (Supplemental Figure 5O). These findings indicate that this macrophage reprogramming mechanism is conserved across murine and human TNBC systems, underscoring its translational relevance.

### TREM2 macrophages mediate resistance against combined therapy.

To investigate the role of TREM2 in macrophage-mediated therapeutic resistance, we employed a TREM2-KO model (C57BL/6J-Trem2^em2Adiuj^/J). BMDMs from TREM2-KO mice demonstrated significantly reduced TREM2 expression on the cell surface and decreased expression of the mC0 macrophage signature genes at both the RNA and protein levels (Supplemental Figure 6, A–C). Furthermore, these macrophages exhibited diminished phagocytosis in vitro (Supplemental Figure 6D).

**Figure 6:**
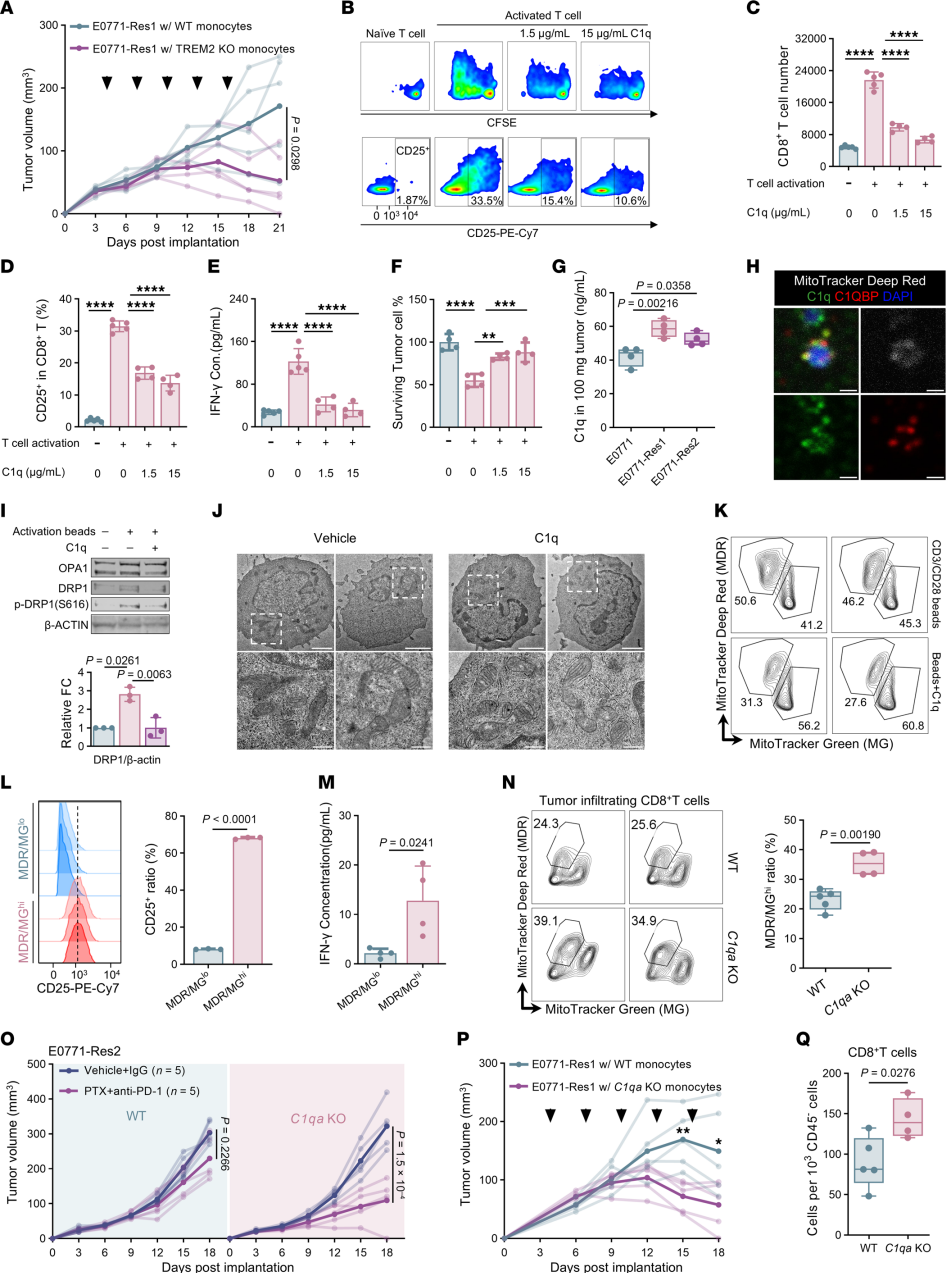
C1q disrupts CD8^+^ T cell metabolism, enabling immune evasion in resistant tumors. (**A**) Growth of E0771-Res1 tumor in CCR2-KO mice receiving WT or TREM2-KO monocytes, under combined therapy (*n* = 5). Arrowheads indicate monocyte transfers. (**B**) Representative flow plots showing CFSE dilution and CD25 expression in CD8^+^ T cell treated with activation beads and indicated concentrations of murine C1q. (**C**–**E**) Quantification of total CD8^+^ T cells (**C**), CD25^+^/total CD8^+^ T cell ratios (**D**), and IFN-γ production by CD8^+^ T cells (**E**). (**F**) Quantification of surviving E0771-OVA cells after coculture with pretreated OT-1 CD8^+^ T cells. (**G**) ELISA quantification of C1q in tumor lysates from E0771, E0771-Res1, and E0771-Res2 tumors (*n* = 4). (**H**) Immunofluorescence of MitoTracker Deep Red, C1q, C1QBP, and DAPI in CD8^+^ T cells. Scale bar: 1 μm. (**I**) Immunoblot of OPA1, DRP1, and phosphor-DRP1 (Ser616) in CD8^+^ T cells. (Bottom) Quantification of DRP1 levels normalized to β-ACTIN (3 independent experiments). (**J**) TEM images of activated CD8^+^ T cells treated with vehicle or C1q for 48 hours. Scale bar: 2 μm; 0.5 μm (zoomed-in). (**K**) Representative flow plots of MitoTracker Deep Red/Green (MDR/MG) populations. (**L** and **M**) CD25 expression (**L**) and IFN-γ production (**M**) in MDR/MG^lo^ versus MDR/MG^hi^ CD8^+^ T cells. (**N**) Representative plots and quantification of MDR/MG^hi^ CD8^+^ T cells from E0771-Res1 tumors in WT (*n* = 5) or *C1qa*-KO mice (*n* = 4). (**O**) Growth of E0771-Res2 tumors in WT or *C1qa*-KO mice under vehicle or combined therapy. The values 0.2266 and 1.5 × 10^4^ indicate represent *P* values for the comparison of tumor volumes between the combined paclitaxel and anti–PD-1 treatment group and the vehicle control at day 18, in wild-type and C1qa knockout mice, respectively. (**P**) Tumor growth of E0771-Res1 tumors in CCR2-KO mice receiving WT or *C1qa*-KO monocytes under combined therapy (*n* = 5). (**Q**) Flow cytometry of CD8^+^ T cells from tumors in **P**. Significance was calculated using 1-way ANOVA followed by Tukey’s test (**C**–**G**, and **I**); unpaired 2-tailed Student’s *t* test (**A** and **L**–**Q**). *P < 0.05, **P < 0.005, ***P < 0.001, ****P < 0.0001.

In vivo, TREM2 KO did not alter peripheral blood immune cell composition in tumor-bearing mice. All major immune populations maintained similar levels between WT and KO mice (Supplemental Figure 6, E and F), including the late-stage monocytes (Supplemental Figure 6G). When treated with the combined treatment, both E0771-Res1 and AT3-Res tumors in the TREM2-KO model exhibited improved response to the therapy (Supplemental Figure 6H). Furthermore, TREM2 KO enhanced immune cell infiltration relative to CD45^–^ cells after treatment (Supplemental Figure 6, I–K), with notably higher levels of CD8^+^ T cells compared with those in WT mice. To directly assess the contribution of TREM2-expressing macrophages, we adoptively transferred WT or TREM2-KO monocytes into CCR2-deficient hosts bearing E0771-Res1 tumors. None of the mice receiving WT monocytes achieved complete tumor regression, whereas mice receiving TREM2-KO monocytes demonstrated significantly improved responses to the combined therapy, with 20% achieving complete regression (Figure 6A). Together, our data indicate that TREM2 expression in TAMs is a driver of acquired resistance to combined chemoimmunotherapy in TNBC.

### TAM-derived C1q suppresses CD8^+^ T cell function.

Based on previous human studies and our data in mice, TREM2^+^ macrophages exhibited elevated expression of *C1q* genes (41, 42) (Figure 3F), and TREM2 KO results in reduced *C1q* expression (Supplemental Figure 6C). Given the established role of C1q deficiency in the inadequate clearance of apoptotic cells and the consequent development of autoimmunity (43), we investigated the involvement of C1q in therapeutic resistance mediated by mC0 macrophages. *C1qa*-KO macrophages (44) lacked C1q expression (Supplemental Figure 7A) and displayed a significantly reduced ability to clear cell debris. This deficiency was restored by supplementing C1q proteins (Supplemental Figure 7B). In WT macrophages, C1q addition further increased the uptake of fluorescent signal, corroborating its role in apoptotic cell recognition (45). Moreover, *C1qa*-KO macrophages exhibited a reduced ability to produce RvD1 in culture and in tumor (Supplemental Figure 7C). These data underscore the importance of C1q in the differentiation and functional capacity of mC0 TREM2^+^C1q^+^ macrophages.

In autoimmune diseases, C1q deficiency provokes an exaggerated effector CD8^+^ T cell response (46). We therefore explored whether TAM-derived C1q could modulate CD8^+^ T cell functions. C1q treatment reduced CD8^+^ T cell proliferation and activation in a dose-dependent manner (Figure 6, B–D) and induced the expression of exhaustion and anergy markers, leading to decreased IFN-γ production (Figure 6E and Supplemental Figure 7D). This resulted in a diminished ability of the T cells to kill cocultured tumor cells (Figure 6F). Similarly, human C1q protein also inhibited human CD8^+^ T cell proliferation, albeit to a lesser extent (Supplemental Figure 7E).

We further assessed the effect of macrophage-produced C1q on CD8^+^ T cell functionality. We confirmed that WT macrophages secreted C1q into the culture medium and that C1qa KO halted C1q production (Supplemental Figure 7F). CD8^+^ T cell survival decreased when treated with WT macrophage culture medium (Supplemental Figure 7G), and IFN-γ production dropped from 5.16 to 3.43 pg/mL (Supplemental Figure 7H). These findings suggest that macrophage-derived C1q plays a crucial role in suppressing CD8^+^ T cell function.

Because monocytes are the chief C1q source (47) and increased circulating monocytes accompany resistant tumors, we asked whether plasma C1q rises systemically. Although plasma C1q rose modestly during tumor growth (Supplemental Figure 7I), levels did not differ between parental- and resistant-tumor hosts (Supplemental Figure 7J). We therefore assessed C1q within the TEM. Notably, C1q levels in resistant tumor lysates were significantly higher than in parental tumors (Figure 6G). These findings indicate that C1q upregulation is confined to the TEM and does not reflect a systemic increase.

### C1q disrupts CD8^+^ T cell metabolism, enabling immune evasion in resistant tumor cells.

scRNA-seq analysis revealed higher expression of the *C1qbp* gene, encoding the mitochondrial-localized C1q receptor, in CD8^+^ T cells (Supplemental Figure 7K) (48). Exogenous C1q added to activated CD8^+^ T cells colocalized with C1QBP inside mitochondria (Figure 6H). During physiological activation, T cell mitochondria undergo DRP1-dependent fission (49), characterized by increased expression of the fission factor DRP1 and its phosphorylation at Ser616 without changes in the fusion mediator OPA1 (Figure 6I). C1q supplementation blunted both DRP1 expression and phosphorylation, yielding swollen mitochondria with sparse cristae and compromised membranes (Figure 6J).

Activation of CD8^+^ T cells led to an increase in mitochondrial membrane potential (Supplemental Figure 7L), which was abrogated by C1q supplementation. We then assessed mitochondrial mass and membrane potential in activated CD8^+^ T cells using MitoTracker Green (MG) and MitoTracker Deep Red (MDR). The MDR/MG ratio reflects mitochondrial activity normalized to mitochondrial mass (50). Consistently, C1q treatment reduced mitochondrial activity in activated CD8^+^ T cells (Figure 6K), resulting in impaired oxidative phosphorylation, diminished T cell activation, and reduced IFN-γ production (Supplemental Figure 7M and Figure 6, L and M).

Furthermore, we evaluated tumor-infiltrating CD8^+^ T cell activity in both WT and *C1qa*-KO mice. The absence of C1q led to a higher proportion of activated CD8^+^ T cells in resistant tumors (Figure 6N). KO of *C1qa* resensitized the resistant tumor to the combined therapy (Figure 6O and Supplemental Figure 7N). Additionally, *C1qa* KO led to increased immune cell infiltration relative to CD45^–^ cells, particularly elevating T cell numbers beyond those seen in the treatment group of WT counterparts (Supplemental Figure 7, O–Q).

To confirm the role of C1q-producing macrophages, we adoptively transferred WT or *C1qa*-KO monocytes into CCR2-deficient hosts bearing tumors. Both grafts infiltrated tumors comparably (Supplemental Figure 7, R and S). Whereas WT monocytes modestly accelerated tumor growth, *C1qa*-KO monocytes failed to confer resistance, resulting in 20% complete tumor regression and a marked increase in CD8^+^ T cell infiltration relative to CD45^–^ cells (Figure 6, P and Q).

Taken together, our findings reveal that therapy-resistant, macrophage-rich TNBC tumors with low inflammation incorporate unsaturated fatty acids into their membranes, stimulate resolvin production via macrophage efferocytosis, and drive monocyte differentiation into TREM2^+^, C1q-secreting macrophages. The resulting C1q cripples mitochondrial dynamics and metabolism in CD8^+^ T cells, curtailing their effector function and permitting immune escape.

### Elongase-5 as a therapeutic target to resensitize resistant TNBC to chemoimmunotherapy.

Elevated polyunsaturated fatty acids (PUFAs) contribute to therapeutic resistance in both proinflammatory and antiinflammatory tumor contexts, but the underlying mechanisms diverge. In inflammatory tumors, resistant cancer cells accumulate triglycerides enriched with AA within LDs, whereas in antiinflammatory tumors, resistance is mediated by the incorporation of ω-3 PUFAs into the cell membrane. In both scenarios, elongase-5 (ELOVL5) is essential for the synthesis and elongation of these PUFAs (Supplemental Figure 8A). Knockdown of *Elovl5* in 2208L-resistant derivatives reduced AA levels and subsequent PGE2 production (Supplemental Figure 8, B and C), thereby enhancing sensitivity to combined chemoimmunotherapy (Supplemental Figure 8D).

**Figure 7:**
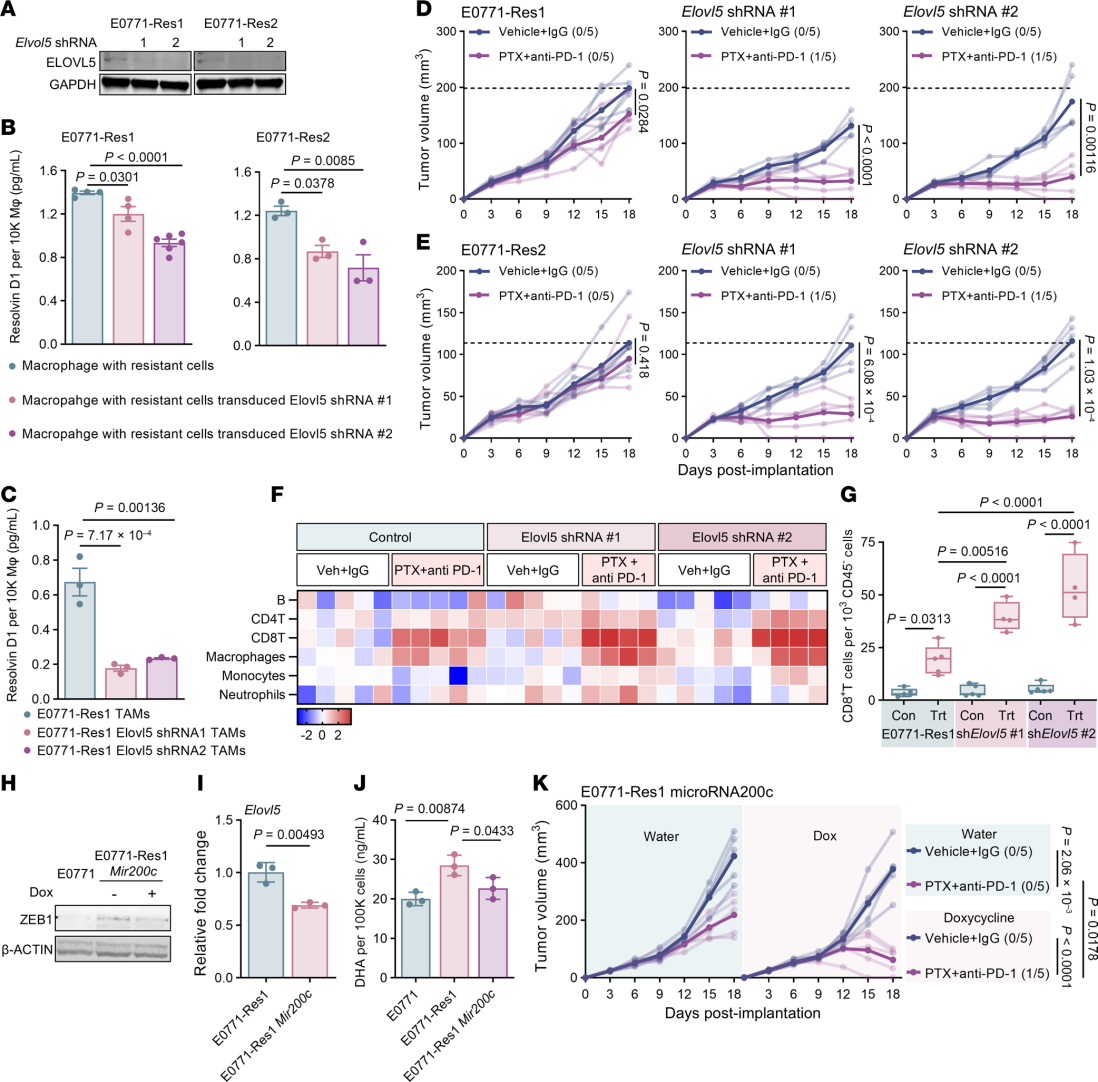
Knockdown of ELOVL5 resensitizes resistant tumor to chemoimmunotherapy. (**A**) Immunoblot of ELOVL5 protein expression in E0771-Res1 and E0771-Res2 cells with *Elvol5* shRNA transduction. GAPDH serves as loading control. (**B**) ELISA quantification of RvD1 in BMDMs cocultured with PTX-treated E0771-Res1 or E0771-Res2 cells transduced with control, *Elovl5* shRNA#1, or shRNA#2. (**C**) ELISA analysis of RvD1 in TAMs isolated from E0771-Res1 cells transduced with control, *Elovl5* shRNA#1, or shRNA#2 tumors. Each data point represents TAMs isolated from an individual tumor (*n* = 3). (**D** and **E**) Growth of E0771-Res1 tumor (**D**), E0771-Res2 tumor (**E**), and their derivatives with *Elovl5* shRNA under vehicle or combined treatment. Significance was calculated using unpaired 2-tailed Student’s *t* test. (**F**) Flow cytometry of immune infiltrates in E0771-Res1 tumors transduced with control, *Elovl5* shRNA#1, or shRNA#2 at endpoint. Log_2_ fold change relative to the average cell number of the vehicle-treated tumor group is displayed. Each square represents a cell type within an individual tumor. (**G**) Quantification of CD8^+^ T cells from tumors in **D**. (**H**) Immunoblot of ZEB1 expression in E0771, E0771-Res1, and E0771-Res1 cells with induced microRNA200c expression. β-ACTIN served as loading control. (**I**) Relative murine *Elovl5* expression in E0771-Res1 cells without or with microRNA200c induction (*n* = 3). Statistical significance was calculated using unpaired 2-tailed Student’s *t* test. (**J**) ELISA quantification of DHA in E0771, E0771-Res1, and E0771-Res1 cells with induced microRNA200c expression (*n* = 3). (**K**) Tumor growth of E0771-Res1 without or with microRNA200c induction under control or PTX plus anti–PD-1 antibody treatment. Numbers indicate cured/total mice in each group. Statistical significance of tumor volume at day 18 was assessed using 1-way ANOVA followed by Tukey’s test. (**B**, **C**, **G**, and **J**) Statistical significance was determined using 1-way ANOVA followed by Tukey’s test.

Similarly, knockdown of *Elovl5* in E0771-resistant cell lines markedly reduced RvD1 production by cocultured macrophages in vitro (Figure 7, A and B) and by TAMs in vivo (Figure 7C). *Elovl5* knockdown restored therapeutic responsiveness in both resistant tumor models, leading to complete tumor regression in some mice (Figure 7, D and E) and increasing immune cells infiltration relative to CD45^–^ cells (Figure 7F and Supplemental Figure 8E), especially CD8^+^ T lymphocytes (Figure 7G). Thus, ELOVL5 may represent a therapeutic target for both inflammation- and resolution-driven immunosuppression.

### ZEB1 regulates Elovl5 expression in mesenchymal-like TNBC.

A study by Schwab et al. reported that the EMT transcription factor ZEB1 regulates *Elovl5* expression (51). And E0771-resistant cells displayed elevated expression of mesenchymal markers — particularly *Zeb1* — and correspondingly higher *Elovl5* expression (Supplemental Figure 8, F–H). To determine whether tumor-intrinsic EMT programs directly influence macrophage polarization, we incorporated a doxycycline-inducible microRNA-200c (*Mir200c*) system to modulate EMT status. Doxycycline-induced *Mir200c* expression suppressed ZEB1 in E0771-Res1 cells (Figure 7H), which in turn reduced *Elovl5* expression (Figure 7I) and decreased DHA abundance (Figure 7J). Furthermore, resistant cells secreted substantially higher levels of VEGF, and *Mir200c* induction reversed this increase (Supplemental Figure 8I), thereby diminishing RAW264.7 macrophage recruitment in a Trans-well assay (Supplemental Figure 8J).

Finally, to assess the functional consequence of reversing ZEB1 expression in vivo, we evaluated whether *Mir200c* induction could resensitize resistant tumors to combined therapy. Doxycycline-mediated *Mir200c* expression enhanced treatment responsiveness in E0771-Res1 tumors, with complete tumor eradication achieved in 20% of the mice (Figure 7K).

## Discussion

Inflammatory and antiinflammatory (or proresolution) macrophages are enriched in distinct subsets of posttreatment residual tumors, suggesting that they are both associated with therapeutic resistance, albeit in different tumor contexts. This finding appears counterintuitive, given the functionally opposing roles of these macrophage populations. Our data indicate that the antiinflammatory macrophages actively promote immunosuppression and resistance to chemoimmunotherapies. In contrast, the enrichment of inflammatory macrophages may merely reflect an excessively inflammatory microenvironment wherein neutrophils predominantly drive immunosuppression instead (30). Supporting this hypothesis, depletion of macrophages in E0771 and T12, two macrophage-enriched, mesenchymal models, significantly reversed the resistance (Figure 4D and Supplemental Figure 4A), whereas in 2208L, a neutrophil-enriched, epithelial model, the same depletion failed to overcome therapeutic resistance (Supplemental Figure 4H). Additional data reinforced the distinction between neutrophil-driven inflammation and macrophage-driven resolution pathways, including the discrepant effects of NSAID and lipid-droplet dissociation (by perilipin proteins KD) in different models.

Mechanistically, elevated PUFAs contribute to therapeutic resistance in both macrophage-enriched mesenchymal-like and neutrophil-enriched epithelial tumors. However, in the former scenario, resistant cancer cells accumulate ω-3 PUFA, and engulfment of cancer cell debris by macrophages leads to increased production of resolvins (Figure 5, E–I), which in turn promotes differentiation of TREM2^+^ macrophages. Parallelly in the latter scenario, resistance is driven by tumor-derived AA (a ω-6 PUFA) that is transmitted to neutrophils and metabolized into inflammatory mediators (30). Thus, depending on the dominant species of myeloid cells in the TME, TNBC may adopt completely distinct resistant mechanisms that entail different remedies. Interestingly, the epithelial environment, neutrophil recruitment/enrichment, and inflammation mediator production are characteristic of the inflammation phase of wound healing. In contrast, EMT, monocyte recruitment, TREM2^+^ macrophage differentiation, and resolvin generation mirror the resolution phase of wound healing. Therefore, tumors are indeed unhealing wounds stalled at different stages. Importantly, the selective pressure of chemoimmunotherapies further accentuates these phenotypic divergences.

These parallel mechanisms imply different strategies to overcome the therapeutic resistance. However, translating these approaches clinically may remain challenging without reliable biomarkers to differentiate patient subtypes. The neutrophil-enriched tumors are accompanied by systemic neutrophil accumulation (29, 52), which may serve as a diagnostic indicator. Alternatively, common regulators of both ω-3 and ω-6 PUFAs may represent novel therapeutic targets that can mitigate both inflammation and inflammation resolution. ELOVL5 is a promising candidate in this regard based on our data (Figure 7 and Supplemental Figure 8D and Yu et al., ref. 30). To our knowledge, no pharmacologically favorable ELOVL5 inhibitors exist, highlighting a potential avenue for future research (Supplemental Figure 8K).

ω-3 PUFAs have been reported to reduce cancer risk (53), and in vitro studies suggest their potential to enhance the efficacy of chemotherapy, including PTX, doxorubicin, and tamoxifen (54–56). Proposed mechanisms underlying ω-3 PUFAs-induced chemosensitizing effects include (a) reducing eicosanoid synthesis from AA, which alters immune responses within the TME (57); (b) modulating cellular proliferation via the Akt/NF-κB pathway (58); (c) inducing apoptosis through the production of reactive oxygen species (59); and (d) impairing multidrug-resistant transporter functions by altering membrane lipid composition (60). However, a systematic meta-analysis revealed no significant association between ω-3 PUFA intake and cancer incidence (61). Berquin et al. demonstrated that a lower ω-6 to ω-3 fatty acid ratio effectively slows cancer progression (62), whereas elevated ω-6 PUFA intake promotes tumor progression in both animal and human models (63). Despite these observations, the comprehensive effects of PUFAs on chemoimmunotherapies are only beginning to be elucidated.

## Methods

### Sex as a biological variable.

Our study exclusively examined female mice since the disease modeled is mainly relevant in females.

### Animal models.

BALB/c (model 047) and C57BL/6 (model 044) mice were purchased from Envigo. B6.129S4-Ccr2^tm1Ifc^/J (strain 004999), C57BL/6J-Trem2^em2Adiuj^/J (strain 027197), B6(Cg)-C1qa^tm1d(EUCOMM)Wtsi^/TennJ (strain 031675), C57BL/6-Tg(TcraTcrb)1100Mjb/J (strain 003831), B6.SJL-Ptprca Pepcb/BoyJ (strain 002014), C57BL/6J-Ms4a3^em2(cre)Fgnx^/J (strain 036382), B6.Cg-Gt(ROSA)26Sor^tm14(CAG-tdTomato)Hze^/J (strain 007914), and C57BL/6J mice (strain 000664) were purchased from The Jackson Laboratory. To generate CCR2-KO mice on a BALB/c background, CCR2-KO mice were crossed with WT BALB/c mice for 5 generations.

### Tumor cell lines.

Murine TNBC lines, including 2208L, EMT6, T11, T12, 4T1, AT3, PyMT-M, and PyMT-N, were cultured in DMEM/high glucose medium (HyClone) containing 10% FBS (Gibco) and antibiotics. E0771 was cultured in RPMI 1640 (HyClone) containing 10% FBS, 10 mM HEPES (HyClone) and antibiotics. All cell lines were cultured in a 5% CO_2_ 37°C incubator. 4T1 (catalog CRL-2539), E0771 (catalog CRL-3461), and EMT6 (catalog CRL-2755) were obtained from ATCC, AT3 (catalog SCC178) was purchased from Millipore, and 2208L, T11 and T12 were provided by Jeff Rosen, Baylor College of Medicine. PyMT-M and PyMT-N were developed by Kim et al. (29).

### Breast tumor models and transplantation.

Orthotopic TNBC models were established by transplanting tumor cells into the inguinal mammary fat pad as previously described (30). Cells were implanted at the following doses: 2208L (1.2 × 10^5^), EMT6 (1.2 × 10^5^), T11 (2 × 10^5^), T12 (1 × 10^5^), AT3 (1.2 × 10^5^), E0771 (1.2 × 10^5^) and PyMT-M (1.2 × 10^5^).

For resistant tumor models, recurrent tumors were resected, enzymatically dissociated in dissociation buffer (DMEM with 10%FBS, 1 mg/mL collagenase II, 2 mg/mL dispase II and 100K unit/mL DNase I), mechanically disrupted, and incubated at 37°C for 1 hour. Single-cell suspensions were filtered through a 70 μm cell strainer (Greiner Bio-One). Single cells were then subjected to red blood cell lysis, and tumor cells were isolated using the Mouse Tumor Cell Isolation Kit (Miltenyi Biotec) according to the manufacturer’s protocol.

### Tissue harvest and dissociation.

Blood was collected using microhematocrit capillary tubes (Kimble). Bone marrow cells were obtained by flushing femurs and tibias with FACS buffer, filtered through a 70 μm strainer, centrifuged at 300*g* for 5 minutes, and subjected to red blood cell lysis on ice for 5 minutes. Tumor tissues were harvested and dissociated as described in *Breast tumor models and transplantation*.

### In vivo drug treatment for combination therapy.

Tumor cells were implanted orthotopically on day 0, and mice were randomized prior to treatment initiation on day 3. Anti–PD-1 antibody (200 μg; clone RMP1-14) was administered intraperitoneally, and PTX (200 μg; Alfa Aesar) was delivered via retro-orbital injection. Single-agent or combination treatments were given every 3 days for a total of 6 doses.

For macrophage depletion, mice received anti–M-CSF antibody (500 μg; clone 5A1) on days 2, 7, and 12 after implantation and clodrosome (100 μL; Encapsula NanoSciences) on days 4, 9, and 14.

Control mice received isotype-matched antibodies and control liposomes. All antibodies were obtained from Bio X Cell and administered intraperitoneally. Tumor size was measured by calipers, and volume was calculated using the formula π/6 × width^2^ × length.

### Flow cytometry.

Single-cell suspensions were prepared as described in *Tissue harvest and dissociation*. Cells were blocked with FcR blocker (1:100, clone 2.4G2) for 10 minutes on ice, followed by staining with directly conjugated antibodies for 25 minutes on ice in the dark. After washing, cells were resuspended in FACS buffer containing DAPI (NucBlue Fixed Cell ReadyProbes Reagent) and counting beads (BD Biosciences) and analyzed on a BD LSR Fortessa or LSRII. Data were processed using FlowJo v10.0. The following antibodies (with clone IDs indicated) were used for immune profiling and the myeloid cell panel: CD45-violetFluor450 (30-F11, Cytek), CD11b-APC-Cy7 (M1/70, Cytek), Ly6G-PerCPcy5.5 (1A8, Cytek), Ly6C-PE-CF594 (AL-21, BD Biosciences), F4/80-FITC (BM8.1, Cytek), and TREM2-APC (237920, R&D Systems). Antibodies used for the lymphoid cell panel included CD45-violetFluor450 (Cytek), B220-APC-Cy7 (RA3-6B2, Cytek), CD3e-PerCPcy5.5 (145-2C11, Cytek), CD4-APC (GK1.5, Cytek), CD8-FITC (5H10-1, Cytek), and PD-1-PE (RMP1-14, BioLegend). Antibodies used for the T cell exhaustion and anergy panel included CD45-violetFluor450 (30-F11, Cytek), CD3e-PerCPcy5.5 (145-2C11, Cytek), CD8-FITC (Cytek), TIM-3-APC (RMT3-23, Cytek), and PD-1-PE-Dazzle 594 (29F.1A12, BioLegend). Other antibodies used for analysis included CD25-PE-Cy7 (3C7, BioLegend), CD206-AF700 (C068C2, BioLegend), I-A/I-E-BV510 (M5/114.15.2, BioLegend), CD45.1-PE-Cy7 (A20, Cytek), and CD45.2-violetFluor450 (104, Cytek).

### Fluorescence-activated cell sorting and library preparation for scRNA-seq.

Single-cell suspensions from tumors, bone marrow, or peripheral blood were generated as described in *Tissue harvest and dissociation*. Cells were sorted on a FACSAria I or II (BD Biosciences) to isolate monocytes (DAPI^–^/CD45^+^/CD11b^+^/Ly6G^–^/Ly6C^hi^), macrophages (DAPI^–^/CD45^+^/CD11b^+^/Ly6G^–^/Ly6C^–^/F4/80^+^), CD8^+^ T cells (DAPI^–^/CD45^+^/CD3e^+^/CD8a^+^), or total tumor-infiltrating immune cells (DAPI^–^/CD45^+^).

For scRNA-seq, CD45^+^ cells from E0771, E0771-Res1, and E0771-Res2 tumors were labeled with Cell Multiplexing Oligos (10x Genomics), pooled, and processed by the BCM Single Cell Genomics Core. Libraries were generated using the Chromium Single Cell 3′ v3.1 kit (10x Genomics) and sequenced on an Illumina NovaSeq 6000.

### qPCR.

RNA samples were harvested and cDNA were synthesized as previously described (30). Quantitative real-time PCR was carried out using a CFX Real-Time PCR system (Bio-Rad) and PowerUp SYBR Green Master Mix (Thermo Fisher Scientific). Primer sequences are listed in Supplemental Table 1.

### Preprocessing of scRNA-seq dataset.

scRNA-seq data were preprocessed as previously described (30) and analyzed using Seurat v5.2.1 (64, 65) in R v4.1.0. Cells with 500–6,000 detected features and <10% mitochondrial gene content were retained. Mitochondrial (mt-), ribosomal (Rpl/Rps), and Gm-prefixed genes were excluded. Data were normalized using SCTransform, and highly variable genes were used for PCA. UMAP visualization was generated from the top 50 principal components, and cell clustering was performed using SNN-based FindNeighbors and FindClusters functions. Resolution scores were calculated using AUCell (an R-package to analyze the state of gene-sets in single-cell RNA-seq data) based on a curated antiinflammatory macrophage gene signature derived from Patterson et al. (35). The complete gene list used for this signature is provided in Supplemental Table 2.

### RNA velocity analysis in myeloid lineage.

RNA velocity analysis was performed using mapped transcriptomic BAM files. Spliced and unspliced transcript counts were generated with velocyto (66). These velocity matrices were subsequently integrated with transcriptomic data to predict cell state transitions. Cellular trajectories and state transitions were inferred using vector field functions implemented in Dynamo (67) and visualized on myeloid UMAP embeddings.

### scRNA-seq analysis of treatment naive patient samples.

The scRNA-seq dataset was generated by Xu et al. (34) and includes 29 primary treatment-naive TNBC samples. Raw data were processed using Cell Ranger, with initial cell-type annotations adopted from the original study. Patients were stratified into high- and low-resolution groups based on macrophage resolution scores calculated using UCell (68). Hierarchical clustering (K = 2) was performed on per-patient macrophage UCell scores using hclust(), with dendrogram visualization via dendextend (69). Macrophage heterogeneity was assessed by UMAP visualization of macrophage subclusters in Seurat using a clustering resolution of 1.2. The patient IDs and corresponding sample numbers used in Figure 1, I and K, are listed in Supplemental Table 3.

### Western blotting.

Cells were processed as previously described (30). Proteins were loaded on NuPAGE precast gels (Invitrogen) and transferred to nitrocellulose membranes. Membranes were blocked in 5% non-fat milk, incubated with primary antibodies overnight at 4°C, followed by secondary antibodies (LI-COR) for 1 hour at room temperature, and imaged using an Odyssey Fc Imager (LI-COR).

### In situ staining of immune cells in mouse tumors.

Tumor sections were processed as previously described (30), blocked in PBS containing 10% sheep serum and 0.2% Triton X-100 for 45 minutes, and incubated overnight at 4°C with primary antibodies against mouse MPO (1:100, R&D Systems), cleaved caspase-3 (1:200, Cell Signaling), F4/80 (1:200, Cell Signaling), TREM2 (1:200, R&D Systems), RFP (1:200, Rockland Immunochemicals), C1q (1:200, Abcam), and C1QBP (1:200, Santa Cruz). Slides were incubated with appropriate Alexa Fluor–conjugated secondary antibodies (1:400, Jackson ImmunoResearch) for 1 hour at room temperature. Slides were subsequently stained with DAPI. Washing was performed in PBS between all steps.

For immunohistochemistry, sections were incubated with biotin-SP–conjugated secondary antibodies (1:500, Jackson ImmunoResearch), followed by Elite ABC reagent (Vector Laboratories) and DAB development. Nuclei were counterstained with Harris-modified hematoxylin, and sections were dehydrated, cleared, and mounted. All antibodies used in this study are listed in Supplemental Table 4.

### Confocal imaging and image processing.

Ex vivo imaging was performed on Zeiss LSM 780 or LSM 880 inverted confocal microscopes equipped with environmental control chambers. Images were acquired using ZEN software with 16× line averaging, a 40 μm pinhole, and a resolution of 2,048 × 2,048, using 20× and 63× objectives. Image quantification and analysis were conducted using ImageJ (NIH).

### Untargeted lipidomics.

Samples were harvested and processed as previously described (30) and analyzed at the Metabolomics Core Facility of MD Anderson Cancer Center. Raw data were processed and annotated using Thermo Scientific LipidSearch software (v5.1), followed by downstream data analysis using the lipidr package (v2.15.1) (70).

### ELISA.

For ELISA-based quantification of mouse RvD1, RvE1, and LXA4, cells were counted and lysed in 10× ELISA buffer, followed by dilution in ultrapure water. Lysates were centrifuged at 5,000*g* for 10 minutes, and supernatants were collected for analysis. Analyte levels were measured according to the manufacturers’ instructions (Cayman, 500380, 502150, and 590410), with absorbance read at 405 nm using an AccuSkan microplate photometer (Fisher Scientific).

For C1q ELISA assays, plasma was collected by drawing 75 μL blood into tubes containing 1.5 U heparin, centrifuged, and diluted 1:1,000 in diluent buffer. Cell culture supernatants were centrifuged before analysis. Tumor tissues were homogenized in lysis buffer (1 g tissue per 6 mL buffer), incubated for 30 minutes, centrifuged at 5,000*g* for 10 minutes, and supernatants were diluted 1:1 in diluent buffer. C1q levels were quantified according to the manufacturer’s instructions (ab291069, Abcam).

### Statistics.

Mice were randomized after tumor inoculation for treatments. For in vitro experiments on freshly prepared primary tissues/cells, the number of biological replicates in each group was determined by the number of independent donor animals and is indicated in figures. For Western blots, representative data from at least 3 independent experiments are presented. The group sizes were determined based on the results of our previous experiments, and no statistical method was used to predetermine the sample size. All biologically independent samples were included for statistical analyses. Data were quantified using Microsoft Excel and Graphpad v8.0. Data are expressed as mean ± SEM unless otherwise stated. Comparisons between 2 groups were analyzed using unpaired or paired 2-tailed Student’s *t* test. Multiple corrections were analyzed using 1-way ANOVA with Tukey’s post hoc test. Nonparametric data were analyzed by the Mann-Whitney test. *P* values of less than 0.05 were considered statistically significant.

### Study approval.

All animal experiments were performed following the protocols approved by the Baylor College of Medicine (BCM) Institutional Animal Care and Use Committee.

### Data availability.

A Supporting Data Values file is available in accordance with journal policy. Raw and processed scRNA-seq data have been deposited in the Gene Expression Omnibus under accession GSE294252. Any additional information required to reanalyze the data reported in this paper is available from the lead contact upon request.

## Author contributions

LY and CR contributed equally to this work. XHFZ, LY, and CR developed the concept, designed experiments, and wrote the manuscript. LY performed and analyzed the experiments. FL, CR, and YS performed scRNA-seq and bioinformatic analysis. BW performed and analyzed the lipidomics experiments. JL and DGE contributed to mouse strain management. LW, ZX, and DGE assisted in animal work. HLC, WZ, YG, SW, and XC contributed to manuscript editing. XH, YD, LBD, YHW, TDL and XL performed and contributed to preliminary data collection. XHFZ supervised the research.

## Conflict of interest

The authors have declared that no conflict of interest exists.

## Funding support

This work is the result of NIH funding, in whole or in part, and is subject to the NIH Public Access Policy. Through acceptance of this federal funding, the NIH has been given a right to make the work publicly available in PubMed Central.

- US Department of Defense (grants DAMD W81XWH-16-1-0073 and DAMD W81XWH-20-1-0375 to XHFZ).
- US National Cancer Institute (grants CA183878, CA251950, CA221946, CA227904, CA253533, and P50CA186784 to XHFZ and 1F31CA281063-01A1 to CR).
- Breast Cancer Research Foundation (grant BCRF-24-178 to XHFZ).
- US NIH (grants CA125123 and RR024574 to Cytometry and Cell Sorting Core at BCM and S10OD025240 to Single Cell Genomics Core at BCM).
- Cancer Prevention and Research Institute of Texas (grant RP200504 to Single Cell Genomics Core and RP180672 to Cytometry and Cell Sorting Core).

## Supplementary Material

Supplemental data

Unedited blot and gel images

Supporting data values

## Version 1 02/17/2026

In-Press Preview

## Version 2 04/15/2026

Electronic publication
