# Supplementary material for: Inflammation- and resolution-programmed myeloid circuits govern therapeutic resistance in epithelial and mesenchymal triple-negative breast cancer
Source: J Clin Invest. 2026 Feb 17;136(8):e198815. doi: 10.1172/JCI198815 (PMC13078875; doi:10.1172/JCI198815)
Supplement: Supplemental data [file jci-136-198815-s128.pdf]

Supplemental material

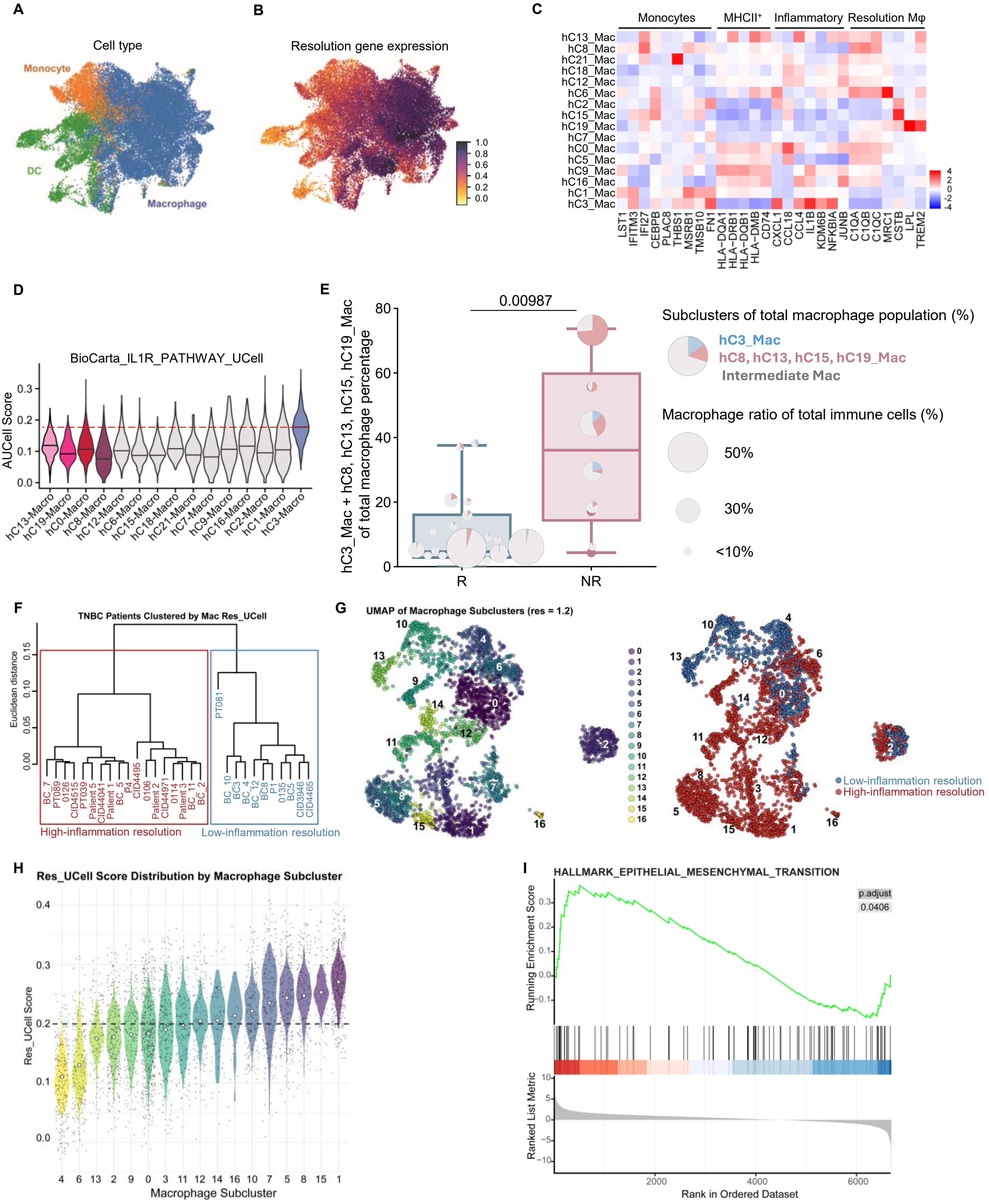

Supplemental Figure 1 Macrophage subclusters in human TNBC patient samples, related to Figure 1

A UMAP plot of myeloid cell clusters.

B UMAP visualization of myeloid cell distribution between responder and non-responders.

C Heatmap illustrates the average gene expression of monocytes, MHCII^{+}, inflammatory, and inflammation resolution macrophage signatures across patient tumor infiltrating macrophage subclusters.

D Violin plots illustrate the BIOCARTA_IL1R_PATHWAY scores across macrophage subclusters, with mean values indicated. Dashed lines denote the corresponding scores for subcluster hC3_Mac.

E Box plots showing the percentages of human macrophage cluster 3 and clusters 8, 13, 15, and 19 in responder and non-responder patients. Each pie chart corresponds to an individual patient, and the pie size reflects the total macrophage fraction among all immune cells within that patient. Statistical significance was assessed using the Mann–Whitney non-parametric test.

F Hierarchical clustering of TNBC patients based on mean macrophage resolution UCell scores. Patients were stratified using hierarchical clustering of their average macrophage resolution scores. Two distinct groups emerged, corresponding to low- (blue) and high-inflammation (red) resolution macrophage profiles. The patient IDs and corresponding sample numbers used in Figures **1I** and **1K** are listed in **Supplemental Table 3**.

G UMAP visualization of macrophage subclusters in TNBC tumors at resolution = 1.2 (Left). Macrophages were clustered using Louvain clustering at resolution 1.2, revealing 17 transcriptionally distinct subclusters (0-16). Each point represents an individual cell, colored by cluster identity. Cluster numbers are labeled in bold for clarity and correspondence with downstream analyses. (Right) UMAP of macrophage subclusters colored by patient resolution group. Cells are color-coded by their patient group: blue for low-inflammation resolution and red for high-inflammation resolution, as defined by mean macrophage UCell scores.

H Distribution of macrophage resolution scores across transcriptional subclusters. Violin plots show the distribution of resolution UCell scores across macrophage subclusters (x-axis), ordered by median score. Each dot represents an individual macrophage cell, and white dots indicate the median score per subcluster. The dashed horizontal line marks the overall mean resolution score across all macrophages.

I Gene Set Enrichment Analysis (GSEA) plot for the HALLMARK_EPITHELIAL_MESENCHYMAL_TRANSITION gene set. GSEA was performed on epithelial cells from TNBC patients with high- vs. low-inflammation resolution scores. The plot shows the running enrichment score (green line) for the EMT hallmark gene set, with vertical black lines indicating the positions of EMT genes within the ranked gene list. The normalized enrichment score was significant with an adjusted p-value of 0.0406, indicating enrichment of EMT-related genes in the high-inflammation resolution group.

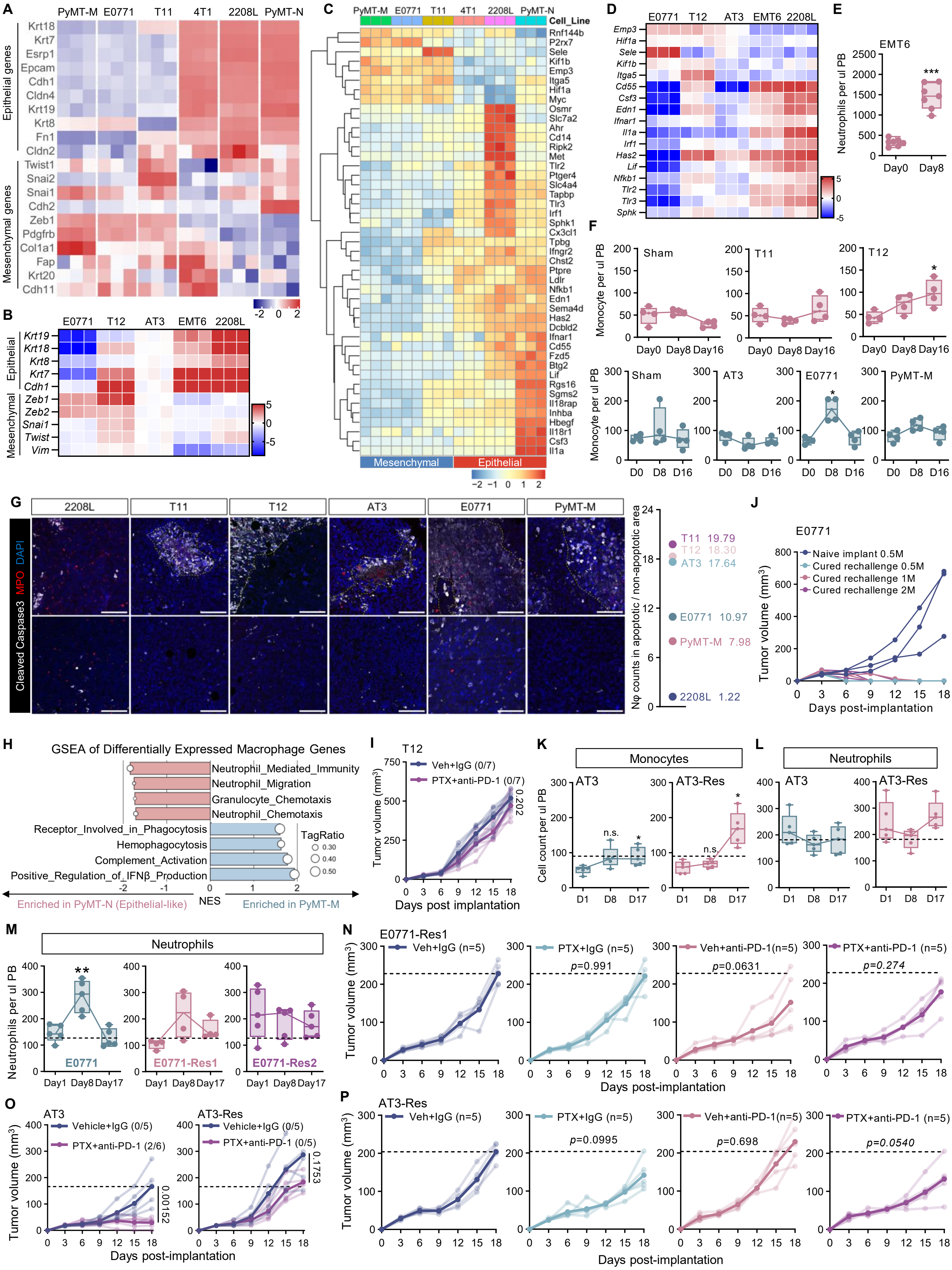

A
PyMT-M E0771 T11 4T1 2208L PyMT-N
Epithelial genes
Krt18
Krt7
Esrp1
Epcam
Cdh1
Cldn4
Krt19
Krt8
Fn1
Cldn2
Mesenchymal genes
Twist1
Snai2
Snai1
Cdh2
Zeb1
Pdgfrb
Col1a1
Fap
Krt20
Cdh11
-2 0 2
B
E0771 T12 AT3 EMT6 2208L
Epithelial
Krt19
Krt18
Krt8
Krt7
Cdh1
Mesenchymal
Zeb1
Zeb2
Snai1
Twist
Vim
5 0 -5
C
PyMT-M E0771 T11 4T1 2208L PyMT-N
Cell_Line
Rnf144b
P2rx7
Sele
Kif1b
Emp3
Itga5
Hif1a
Myc
Osmr
Slc7a2
Ahr
Cd14
Ripk2
Met
Tlr2
Ptger4
Slc4a4
Tapbp
Tlr3
Irf1
Sphk1
Cx3cl1
Tpbg
Ifngr2
Chst2
Ptpre
Ldlr
Nfkb1
Edn1
Sema4d
Has2
Dcbld2
Ifnar1
Cd55
Fzd5
Btg2
Lif
Rgs16
Sgms2
Il18rap
Inhba
Hbegf
Il18r1
Csf3
Il1a
Mesenchymal
Epithelial
−2 −1 0 1 2
D
E0771 T12 AT3 EMT6 2208L
Emp3
Hif1a
Sele
Kif1b
Itga5
Cd55
Csf3
Edn1
Ifnar1
Il1a
Irf1
Has2
Lif
Nfkb1
Tlr2
Tlr3
Sphk
5 0 -5
E
EMT6
Neutrophils per ul PB
2500 2000 1500 1000 500 0
***
Day0 Day8
F
Monocyte per ul PB
Sham T11 T12
200 150 100 50 0
*
Day0 Day8 Day16
Monocyte per ul PB
Sham AT3 E0771 PyMT-M
300 200 100 0
*
D0 D8 D16
G
2208L T11 T12 AT3 E0771 PyMT-M
Cleaved Caspase3 MPO DAPI
Nφ counts in apoptotic / non-apoptotic area
24 18 12 6 0
T11 19.79
T12 18.30
AT3 17.64
E0771 10.97
PyMT-M 7.98
2208L 1.22
J
E0771
Tumor volume (mm3)
800 600 400 200 0
Naive implant 0.5M
Cured rechallenge 0.5M
Cured rechallenge 1M
Cured rechallenge 2M
0 3 6 9 12 15 18
Days post-implantation
H
GSEA of Differentially Expressed Macrophage Genes
Neutrophil_Mediated_Immunity
Neutrophil_Migration
Granulocyte_Chemotaxis
Neutrophil_Chemotaxis
Receptor_Involved_in_Phagocytosis
Hemophagocytosis
Complement_Activation
Positive_Regulation_of_IFNβ_Production
TagRatio
0.30
0.40
0.50
-2 -1 0 1 2
NES
Enriched in PyMT-N (Epithelial-like)
Enriched in PyMT-M
I
T12
Veh+IgG (0/7)
PTX+anti-PD-1 (0/7)
Tumor volume (mm3)
750 500 250 0
0.202
0 3 6 9 12 15 18
Days post implantation
K
Monocytes
AT3 AT3-Res
Cell count per ul PB
300 200 100 0
n.s.
*
n.s.
*
D1 D8 D17
L
Neutrophils
AT3 AT3-Res
400 300 200 100 0
D1 D8 D17
M
Neutrophils
Neutrophils per ul PB
400 300 200 100 0
**
E0771
E0771-Res1
E0771-Res2
Day1 Day8 Day17
N
E0771-Res1
Veh+IgG (n=5)
PTX+IgG (n=5)
p=0.991
Veh+anti-PD-1(n=5)
p=0.0631
PTX+anti-PD-1 (n=5)
p=0.274
Tumor volume (mm3)
300 200 100 0
0 3 6 9 12 15 18
Days post-implantation
O
AT3
Vehicle+IgG (0/5)
PTX+anti-PD-1 (2/6)
0.00152
AT3-Res
Vehicle+IgG (0/5)
PTX+anti-PD-1 (0/5)
0.1753
Tumor volume (mm3)
400 300 200 100 0
0 3 6 9 12 15 18
Days post-implantation
P
AT3-Res
Veh+IgG (n=5)
PTX+IgG (n=5)
p=0.0995
Veh+anti-PD-1(n=5)
p=0.698
PTX+anti-PD-1 (n=5)
p=0.0540
Tumor volume (mm3)
300 200 100 0
0 3 6 9 12 15 18
Days post-implantation

Supplemental Figure 2 Distinct myeloid landscape in murine TNBC models, related to Figure 2.

A Heatmap illustrating the expression of epithelial and mesenchymal marker genes across murine TNBC models, based on bulk RNA-seq data (n=3 per model).

B Heatmap showing the expression of epithelial and mesenchymal marker genes across murine TNBC models measured by real-time PCR (n = 3 per model). The average expression level of AT3 cells was used as the reference baseline.

C Heatmap showing the average expression of genes in the HALLMARK_INFLAMMATORY_ RESPONSE signature across murine TNBC models, based on bulk RNA-seq data.

D Heatmap showing the average expression of genes in the HALLMARK_INFLAMMATORY_ RESPONSE signature across murine TNBC models measured by real-time PCR. The average expression of all five cell lines was used as the reference baseline.

E Flow cytometry analysis of peripheral blood (PB) neutrophils in BALB/c mice with EMT6 tumors (n=7). PB were harvested and analyzed on day 0 and day 8 after tumor implantation.

F Flow cytometry analysis of peripheral blood monocytes in BALB/c mice (pink) with sham (surgery without tumor implantation), T11, T12 tumors, and in C57BL/6 mice (blue) with sham, AT3, E0771, PyMT-M tumors. PB were harvested and analyzed on day 0, 8 and 16 after tumor implantation, with each group consisting of four mice.

G (Left) Representative immunofluorescence staining of cleaved caspase-3, MPO, and DAPI in 2208L, T11, T12, AT3, E0771, and PyMT-M tumors. Scale bar, 100 μm. (Right) Quantification of neutrophil density within apoptotic versus non-apoptotic regions.

H Gene Set Enrichment Analysis of Gene Ontology (GO) pathways based on differentially expressed genes in tumor infiltrating macrophages between PyMT-M (mesenchymal-like) and PyMT-N (epithelial-like) tumors. Pathways with a Benjamini–Hochberg adjusted p value < 0.05 were considered significant. For space reasons, the GO term “GOBP_IMMUNE_RESPONSE_REGULATING_CELL_SURFACE_ RECEPTOR_SIGNALING_PATHWAY_INVOLVED_IN_PHAGOCYTOSIS” was shorted to “RECEPTOR _ INVOLVED_IN_PHAGOCYTOSIS”.

I Tumor growth of T12 under vehicle or paclitaxel (PTX) plus anti-PD-1 antibody. Numbers in parentheses indicate cured mice/total mice in each group. Growth curves of groups (daker lines) and single mouse (lighter lines) are shown.

J Tumor growth of E0771 in treatment-naïve mice and in mice previously cured from E0771 tumors. Treatment-naïve mice were inoculated with 0.5 million E0771 cells, while cured mice were rechallenged with 0.5 to 2 million E0771 cells.

K and **L** Flow cytometry analysis of peripheral blood monocytes (K) and neutrophils (L) in AT3 and AT3-Res tumor bearing mice (n=5) on day 1, 8 and 17 after tumor implantation.

M Flow cytometry analysis of peripheral blood neutrophils in E0771, E0771-Res1 and E0771-Res2 tumor bearing mice (n=5) on day 1, 8 and 17 after tumor implantation.

N Tumor growth of E0771-Res1 tumors treated with vehicle plus control IgG, PTX plus control IgG, vehicle plus anti-PD-1 antibody, and PTX plus anti-PD-1 antibody. Growth curves of groups (daker lines) and single mouse (lighter lines) are shown. Each experimental group includes tumors from five individual mice.

O Tumor growth of AT3 (left) and AT3-Res (right) under vehicle or PTX plus anti-PD-1 antibody. Numbers in parentheses indicate cured mice/total mice in each group. Growth curves of groups (daker lines) and single mouse (lighter lines) are shown.

P Tumor growth of AT3-Res tumors treated with vehicle plus control IgG, PTX plus control IgG, vehicle plus anti-PD-1 antibody, and PTX plus anti-PD-1 antibody. Growth curves of groups (daker lines) and single mouse (lighter lines) are shown. Each experimental group includes tumors from five individual mice.

(**D, I, O**) Significance was calculated using unpaired two-tailed Student's t test.

(**E, F, K-N, P**) Statistical significance was determined using one-way ANOVA followed by Tukey's test.

* $p<0.05$, ** $p < 0.005$, *** $p < 0.001$.

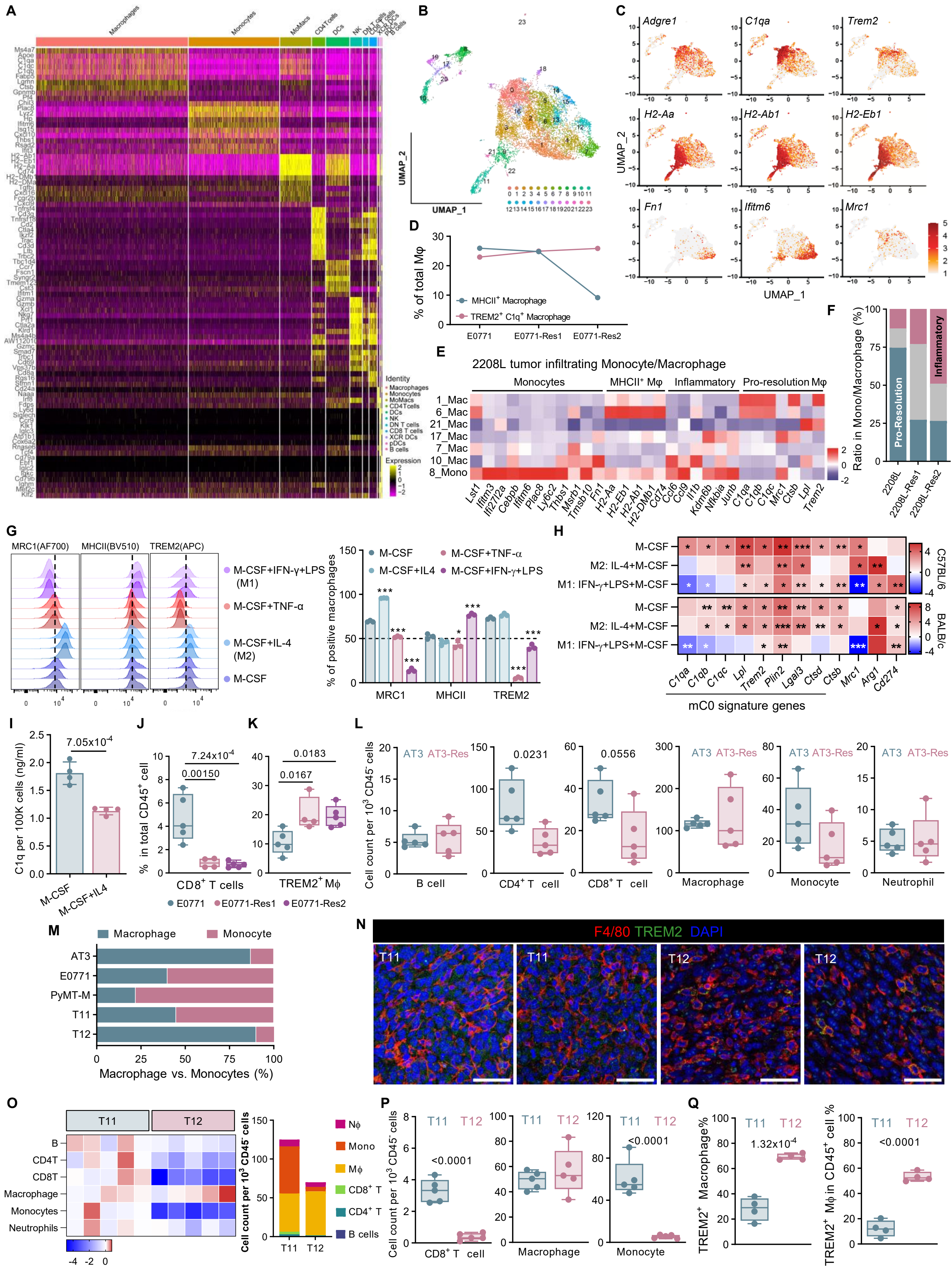

A
Macrophages
Monocytes
MoMacs
CD4Tcells
DCs
NK
Identity
Macrophages
Monocytes
MoMacs
CD4Tcells
DCs
NK
DN T cells
CD8 T cells
XCR DCs
pDCs
B cells
Expression
B
UMAP_2
UMAP_1
C
Adgre1
C1qa
Trem2
H2-Aa
H2-Ab1
H2-Eb1
Fn1
Ifitm6
Mrc1
UMAP_2
UMAP_1
D
% of total Mφ
MHCII+ Macrophage
TREM2+ C1q+ Macrophage
E0771
E0771-Res1
E0771-Res2
E
2208L tumor infiltrating Monocyte/Macrophage
Monocytes
MHCII+ Mφ
Inflammatory
Pro-resolution Mφ
1_Mac
6_Mac
21_Mac
17_Mac
7_Mac
10_Mac
8_Mono
Lst1
Ifitm3
Ifi27l2a
Cebpb
Ifitm6
Plac8
Ly6c2
Thbs1
Msrb1
Tmsb10
Fn1
H2-Aa
H2-Eb1
H2-Ab1
H2-DMb1
Cd74
Ccl6
Ccl9
Il1b
Kdm6b
Nfkbia
Junb
C1qa
C1qb
C1qc
Mrc1
Ctsb
Lpl
Trem2
F
Ratio in Mono/Macrophage (%)
Pro-Resolution
Inflammatory
2208L
2208L-Res1
2208L-Res2
G
MRC1(AF700)
MHCII(BV510)
TREM2(APC)
M-CSF+IFN-γ+LPS (M1)
M-CSF+TNF-α
M-CSF+IL-4 (M2)
M-CSF
M-CSF
M-CSF+IL4
M-CSF+TNF-α
M-CSF+IFN-γ+LPS
% of positive macrophages
MRC1
MHCII
TREM2
H
M-CSF
M2: IL-4+M-CSF
M1: IFN-γ+LPS+M-CSF
C57BL/6
BALB/c
C1qa
C1qb
C1qc
Lpl
Trem2
Plin2
Lgal3
Ctsd
Ctsb
Mrc1
Arg1
Cd274
mC0 signature genes
I
C1q per 100K cells (ng/ml)
7.05x10-4
M-CSF
M-CSF+IL4
J
% in total CD45+ cell
7.24x10-4
0.00150
CD8+ T cells
K
0.0183
0.0167
TREM2+ Mφ
E0771
E0771-Res1
E0771-Res2
L
Cell count per 10³ CD45- cells
AT3
AT3-Res
0.0231
0.0556
B cell
CD4+ T cell
CD8+ T cell
Macrophage
Monocyte
Neutrophil
M
Macrophage
Monocyte
AT3
E0771
PyMT-M
T11
T12
Macrophage vs. Monocytes (%)
N
F4/80 TREM2 DAPI
T11
T11
T12
T12
O
T11
T12
B
CD4T
CD8T
Macrophage
Monocytes
Neutrophils
Cell count per 10³ CD45- cells
Nϕ
Mono
Mϕ
CD8+ T
CD4+ T
B cells
P
Cell count per 10³ CD45- cells
T11
T12
<0.0001
CD8+ T cell
Macrophage
Monocyte
Q
TREM2+ Macrophage%
1.32x10-4
TREM2+ Mϕ in CD45+ cell %
<0.0001

Supplemental Figure 3 Resistant tumors promote tumor associate macrophage differentiation, related to Figure 3.

A Heatmap of top 10 differentially expressed genes across all detected $CD45^+$ immune cell populations.

B UMAP plots of $CD45^+$ cell clusters from merged tumor samples

C Feature plots of *Adgre1*, *C1qa*, *Trem2*, *H2-Aa, H2-Ab1*, *H2-Eb1*, *Fn1, Ifitm6* and *Mrc1* expression level in $CD45^+$ immune cells.

D Percentage of $TREM2^+$ $C1q^+$ macrophages (mC0 in E0771 tumors) and $MHCII^+$ macrophages (mC3) within total macrophage populations.

E Heatmap illustrates the average gene expression of monocytes, $MHCII^+$, inflammatory, and inflammation resolution macrophage signatures across 2208L tumor infiltrating macrophage/monocyte subclusters.

F Ratio of $TREM2^{high}$ macrophages (Cluster 1 in 2208L tumors) and inflammatory macrophages (Cluster 7, 10 and 17 in 2208L tumors) within the combined monocyte and macrophage populations. Grey bars represent all other macrophage clusters defined in Fig. S3E.

G Representative flow plots (left panels) and quantification (right panels) of MRC1, MHCII and TREM2 expression on macrophages stimulated with M-CSF (10 ng/ml), M-CSF plus IL-4 (10 ng/ml), M-CSF plus TNF-α (10 ng/ml) and M-CSF plus IFN-γ (20 ng/ml) and LPS (50 ng/ml). BMDMs were obtained from three individual mice.

H Relative expression of murine Cluster 0 macrophage-specific genes in BALB/c and C57BL/6 BMDMs polarized with the indicated regulators. Same samples from Fig. S3G were analyzed. BMDMs treated with M-CSF plus TNF-α served as the baseline control.

I ELISA quantification of C1q levels in culture medium collected from BMDMs treated with M-CSF alone and M-CSF + IL4 (n=4).

J and **K** Flow cytometry analysis showing the proportions of $CD8^+$ T cells (**J**) and $TREM2^+$ macrophages (**K**) within the total $CD45^+$ immune cell compartment.

L Flow cytometry analysis of major immune cell populations per 1000 $CD45^-$ cells in AT3 and AT3-Res tumors. n = 5 per group.

M Flow cytometry analysis of the ratio of tumor-infiltrating macrophages to monocytes in tumors (AT3, E0771, PyMT-M, T11, and T12) 18 days post-implantation. Each experimental group includes tumors from four individual mice.

N Representative immunofluorescence staining for F4/80, TREM2 and DAPI of T11 and T12 tumors. Scale bar: 50 μm.

O Flow cytometry analysis of the immune infiltrates in T11 and T12 tumors. (Left) Log2 fold change of major immune cells relative to the average cell number of T11 group, with each square representing a specific cell type within an individual tumor. (Right) Average immune cell number per 1000 $CD45^-$ cells. Each experimental group includes tumors from five individual mice.

P Flow cytometry analysis of indicated immune cell populations per 1000 $CD45^-$ cells in T11 and T12 tumors.

Q Flow cytometry analysis of $TREM2^+$ macrophage frequencies within the total macrophage population (left) and within the total $CD45^+$ immune cell compartment (right) in T11 and T12 tumors.

(**G, H, J, K**) Statistical significance was determined using one-way ANOVA followed by Tukey's test.

(**I, L, P, Q**) Significance was calculated using unpaired two-tailed Student's t test.

* $p<0.05$, ** $p < 0.005$, *** $p < 0.001$.

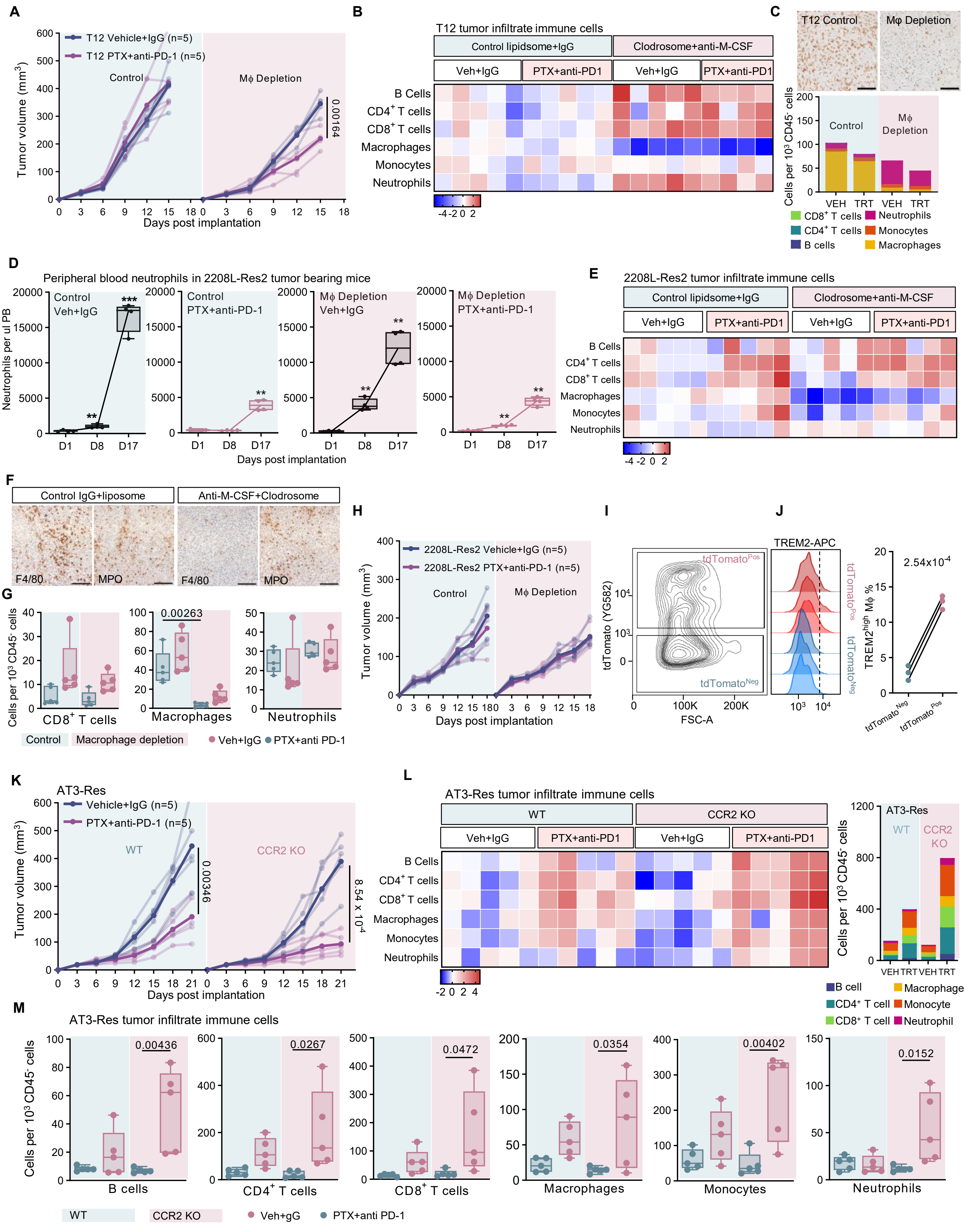

A
T12 Vehicle+IgG (n=5)
T12 PTX+anti-PD-1 (n=5)
Control
Mϕ Depletion
0.00164
Tumor volume (mm3)
Days post implantation
B
T12 tumor infiltrate immune cells
Control lipidsome+IgG
Clodrosome+anti-M-CSF
Veh+IgG
PTX+anti-PD1
B Cells
CD4+ T cells
CD8+ T cells
Macrophages
Monocytes
Neutrophils
C
T12 Control
Mφ Depletion
Cells per 10^3 CD45- cells
Control
Mϕ Depletion
VEH TRT VEH TRT
CD8+ T cells
CD4+ T cells
B cells
Neutrophils
Monocytes
Macrophages
D
Peripheral blood neutrophils in 2208L-Res2 tumor bearing mice
Control Veh+IgG
Control PTX+anti-PD-1
Mϕ Depletion Veh+IgG
Mϕ Depletion PTX+anti-PD-1
Neutrophils per ul PB
Days post implantation
E
2208L-Res2 tumor infiltrate immune cells
Control lipidsome+IgG
Clodrosome+anti-M-CSF
F
Control IgG+liposome
Anti-M-CSF+Clodrosome
F4/80
MPO
G
Cells per 10^3 CD45- cells
CD8+ T cells
Macrophages
Neutrophils
0.00263
Control
Macrophage depletion
Veh+IgG
PTX+anti PD-1
H
2208L-Res2 Vehicle+IgG (n=5)
2208L-Res2 PTX+anti-PD-1 (n=5)
Control
Mϕ Depletion
Tumor volume (mm3)
Days post implantation
I
tdTomatoPos
tdTomatoNeg
tdTomato (YG582)
FSC-A
J
TREM2-APC
TREM2high Mϕ %
2.54x10-4
tdTomatoNeg
tdTomatoPos
K
AT3-Res
Vehicle+IgG (n=5)
PTX+anti-PD-1 (n=5)
WT
CCR2 KO
0.00346
8.54 x 10-4
Tumor volume (mm3)
Days post implantation
L
AT3-Res tumor infiltrate immune cells
WT
CCR2 KO
Veh+IgG
PTX+anti-PD1
AT3-Res
Cells per 10^3 CD45- cells
VEH TRT VEH TRT
B cell
CD4+ T cell
CD8+ T cell
Macrophage
Monocyte
Neutrophil
M
AT3-Res tumor infiltrate immune cells
Cells per 10^3 CD45- cells
0.00436
0.0267
0.0472
0.0354
0.00402
0.0152
B cells
CD4+ T cells
CD8+ T cells
Macrophages
Monocytes
Neutrophils
WT
CCR2 KO
Veh+gG
PTX+anti PD-1

Supplemental Figure 4 Monocyte-derived tumor-associated macrophages are key mediators of therapy resistance, related to Figure 4.

A Tumor growth of T12 under vehicle or combined therapy, with or without macrophage depletion reagents. Each experimental group includes five individual mice.

B Flow cytometry analysis of the immune infiltrates in T12 tumors at the end of experiments. Each square represents a specific cell type within an individual tumor, with log2 fold changes normalized to the average cell number of the control group.

C Analysis of the immune infiltrates in T12 tumors at the end of experiments. (Upper panel) Representative immunohistochemistry staining of F4/80 in T12 tumors following treatment with control IgG plus liposome or anti-M-CSF plus Clodrosome. Scale bar: 100 µm. (Lower panel) Flow cytometry analysis of average immune cell number per 1000 $CD45^-$ cells.

D Flow cytometry analysis of peripheral blood neutrophils in 2208L-Res2 tumor-bearing mice, comparing control treatment and combined therapy with or without macrophage depletion. Samples were collected on day 1, 8 and 17 post-implantation.

E Flow cytometry analysis of the immune infiltrates in 2208L-Res2 tumors at the end of experiments. Each square represents a specific cell type within an individual tumor, with log2 fold changes normalized to the average cell number of the control group.

F Representative immunohistochemistry staining of F4/80 and MPO in 2208L-Res2 tumors following treatment with control IgG plus liposome or anti-M-CSF plus clodrosome. Scale bar: 100 µm.

G Flow cytometry analysis of major immune cell number per 1000 $CD45^-$ cells in 2208L-Res2 tumors under indicated treatments.

H Tumor growth of 2208L-Res2 under vehicle or combined therapy, with or without macrophage depletion reagents. Each experimental group includes five individual mice.

I Flow cytometric analysis of tdTomato expression in tumor-infiltrating macrophages from $Ms4a3^{Cre}$-$Rosa^{TdT}$ mice bearing E0771 tumors.

J Flow cytometry analysis of TREM2 expression in tdTomato^{+} versus tdTomato^{-} tumor-associated macrophages (left; dashed line indicates the gating cutoff), and quantification of TREM2 expression levels in each population (right; n = 3).

K Tumor growth of AT3-Res in wild-type or CCR2 KO mice treated with vehicle or PTX combined with anti-PD-1. Each experimental group includes five individual mice.

L Flow cytometry analysis of the immune infiltrates in AT3-Res tumors at the end of experiments. (Left) Log2 fold change of major immune cells relative to the average cell number of the vehicle-treated tumor group. Each square represents a specific cell type within an individual tumor. (Right) Average immune cell number per 1000 CD45^{-} cells.

M Flow cytometry analysis of major immune cell number per 1000 CD45^{-} cells in AT3-Res tumors in wild-type vs. CCR2 KO mice.

(**A, H, J**) Significance was calculated using unpaired two-tailed Student's t test.

(**D, G, K, M**) Statistical significance was determined using one-way ANOVA followed by Tukey's test.

* $p<0.05$, ** $p < 0.005$, *** $p < 0.001$.

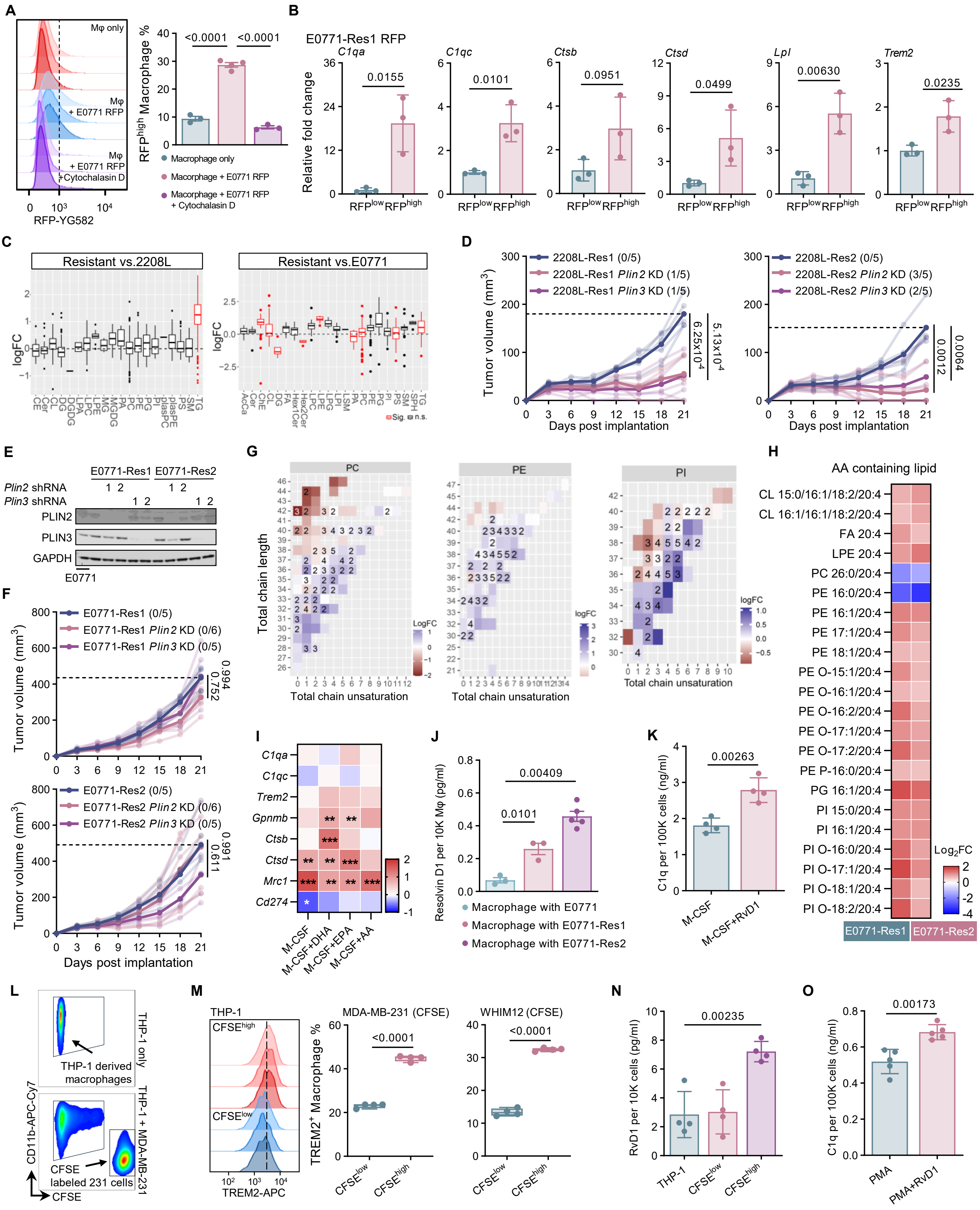

A
Mφ only
Mφ + E0771 RFP
Mφ + E0771 RFP +Cytochalasin D
RFP-YG582
RFPhigh Macrophage %
<0.0001
<0.0001
Macrophage only
Macorphage + E0771 RFP
Macorphage + E0771 RFP + Cytochalasin D
B
E0771-Res1 RFP
Relative fold change
C1qa
0.0155
C1qc
0.0101
Ctsb
0.0951
Ctsd
0.0499
Lpl
0.00630
Trem2
0.0235
RFPlow RFPhigh
C
Resistant vs.2208L
Resistant vs.E0771
logFC
Sig. n.s.
D
Tumor volume (mm3)
2208L-Res1 (0/5)
2208L-Res1 Plin2 KD (1/5)
2208L-Res1 Plin3 KD (1/5)
6.25x10-4
5.13x10-4
2208L-Res2 (0/5)
2208L-Res2 Plin2 KD (3/5)
2208L-Res2 Plin3 KD (2/5)
0.0012
0.0064
Days post implantation
E
E0771-Res1
E0771-Res2
Plin2 shRNA
Plin3 shRNA
PLIN2
PLIN3
GAPDH
E0771
F
E0771-Res1 (0/5)
E0771-Res1 Plin2 KD (0/6)
E0771-Res1 Plin3 KD (0/5)
0.994
0.752
E0771-Res2 (0/5)
E0771-Res2 Plin2 KD (0/6)
E0771-Res2 Plin3 KD (0/5)
0.991
0.611
Tumor volume (mm3)
Days post implantation
G
PC
PE
PI
Total chain length
Total chain unsaturation
LogFC
logFC
H
AA containing lipid
CL 15:0/16:1/18:2/20:4
CL 16:1/16:1/18:2/20:4
FA 20:4
LPE 20:4
PC 26:0/20:4
PE 16:0/20:4
PE 16:1/20:4
PE 17:1/20:4
PE 18:1/20:4
PE O-15:1/20:4
PE O-16:1/20:4
PE O-16:2/20:4
PE O-17:1/20:4
PE O-17:2/20:4
PE P-16:0/20:4
PG 16:1/20:4
PI 15:0/20:4
PI 16:1/20:4
PI O-16:0/20:4
PI O-17:1/20:4
PI O-18:1/20:4
PI O-18:2/20:4
Log2FC
E0771-Res1
E0771-Res2
I
C1qa
C1qc
Trem2
Gpnmb
Ctsb
Ctsd
Mrc1
Cd274
M-CSF
M-CSF+DHA
M-CSF+EPA
M-CSF+AA
J
Resolvin D1 per 10K Mφ (pg/ml)
0.00409
0.0101
Macrophage with E0771
Macrophage with E0771-Res1
Macrophage with E0771-Res2
K
C1q per 100K cells (ng/ml)
0.00263
M-CSF
M-CSF+RvD1
L
THP-1 only
THP-1 derived macrophages
THP-1 + MDA-MB-231
CFSE labeled 231 cells
CD11b-APC-Cy7
CFSE
M
THP-1
CFSEhigh
CFSElow
TREM2-APC
MDA-MB-231 (CFSE)
<0.0001
WHIM12 (CFSE)
<0.0001
TREM2+ Macrophage %
CFSElow
CFSEhigh
N
RvD1 per 10K cells (pg/ml)
0.00235
THP-1
CFSElow
CFSEhigh
O
C1q per 100K cells (ng/ml)
0.00173
PMA
PMA+RvD1

Supplemental Figure 5 Efferocytosis and resolvin production promote TREM2⁺ macrophage differentiation, related to Figure 5.

A Representative flow plots (left, dashed line indicates the gating cutoff) and the quantitative results (right) of RFP signal in BMDMs after co-culture with PTX pre-treated E0771 RFP cells for 24 hours. BMDMs were treated with either vehicle or cytochalasin d (1 ug/ml) during the co-culture.

B Relative expression of murine Cluster 0 macrophages specific genes in FACS sorted BMDMs. BMDMs with or without RFP signal after 24 hours of co-culture with PTX pre-treated E0771-Res1 RFP cells (n=3).

C Lipidomics analysis comparing 2208L (left) and E0771 (right) resistant tumor cells to parental cells. Enrichment analysis of lipid classes highlighted by their relative abundance and significance.

D Tumor growth of 2208L-Res1 (left), 2208L-Res2 (right) transduced with control, *Plin2* shRNA or *Plin3* shRNA under PTX plus anti-PD-1 antibody treatment. Numbers in parentheses indicate cured mice/total mice in each group.

E Immunoblot analysis of PLIN2 and PLIN3 protein expression in E0771 and resistant cells with *Plin2* and *Plin3* shRNA transduction. Glyceraldehyde 3-phosphate dehydrogenase (GAPDH) serves as the loading control.

F Tumor growth of E0771-Res1 (left) and E0771-Res2 (right) transduced with control, *Plin2* shRNA or *Plin3* shRNA under PTX plus anti-PD-1 antibody treatment. Numbers in parentheses indicate cured mice/total mice in each group.

G Visualization of lipid fold changes categorized by total chain length and degree of unsaturation. Numbers represent the count of distinct lipids sharing identical chain properties within each category. Lipid abundance in E0771-Res1 and E0771-Res2 combined was compared against E0771 parental cells.

H Comparative analysis of AA-containing lipids in E0771 resistant cells versus parental cells. Lipids demonstrating an absolute log2 fold change greater than 0.5 and a p-value below 0.05 were included.

I Relative expression of murine Cluster 0 macrophage-specific genes in BMDMs treated with M-CSF at doses of 5 ng/ml and 10 ng/ml, and M-CSF (5 ng/ml) combined with DHA, EPA, or AA (5 μM). BMDMs treated with M-CSF (5 ng/ml) alone served as the control group.

J ELISA quantification of resolvin D1 in BMDMs co-cultured with PTX pre-treated E0771 (n=3), E0771-Res1 (n=3) and E0771-Res2 (n=5) cells. Macrophages were sorted 20 hours after co-culture.

K ELISA quantification of C1q levels in culture medium collected from BMDMs treated with M-CSF alone and M-CSF+RvD1 (n=4).

L Representative flow plots of THP-1 derived macrophages cultured alone (upper panel) or co-culture with paclitaxel pre-treated, CFSE-labeled MDA-MB-231 cells (lower panel).

M Flow cytometry analysis (left) and the quantitative comparison of TREM2 expression levels in $CFSE^{low}$ vs. $CFSE^{high}$ THP-1 derived macrophages after co-culture with paclitaxel pre-treated, CFSE-labeled MDA-MB-231 cells (middle, n=4) or CFSE-labeled WHIM12 cells (right, n=4). Dashed line indicate the APC signal level at which the majority of cells express.

N ELISA quantification of RvD1 in THP-1 derived macrophages co-cultured with paclitaxel pre-treated, CFSE-labeled WHIM12 cells (n=4). Macrophages were sorted based on their CFSE signal prior to analysis. Macrophage cultured alone (n=4) were used as baseline control.

O ELISA quantification of C1q levels in culture medium collected from THP-1 derived macrophages treated with phorbol 12-myristate 13-acetate (PMA) or PMA+RvD1 (n=5).

(**A**, **D**, **F**, **I**, **J**, **N**) Statistical significance was determined using one-way ANOVA followed by Tukey's post hoc test.

(**B**, **K**, **M**, **O**) Statistical significance was calculated using unpaired two-tailed Student's t test.

* $p<0.05$, ** $p < 0.005$, *** $p < 0.001$.

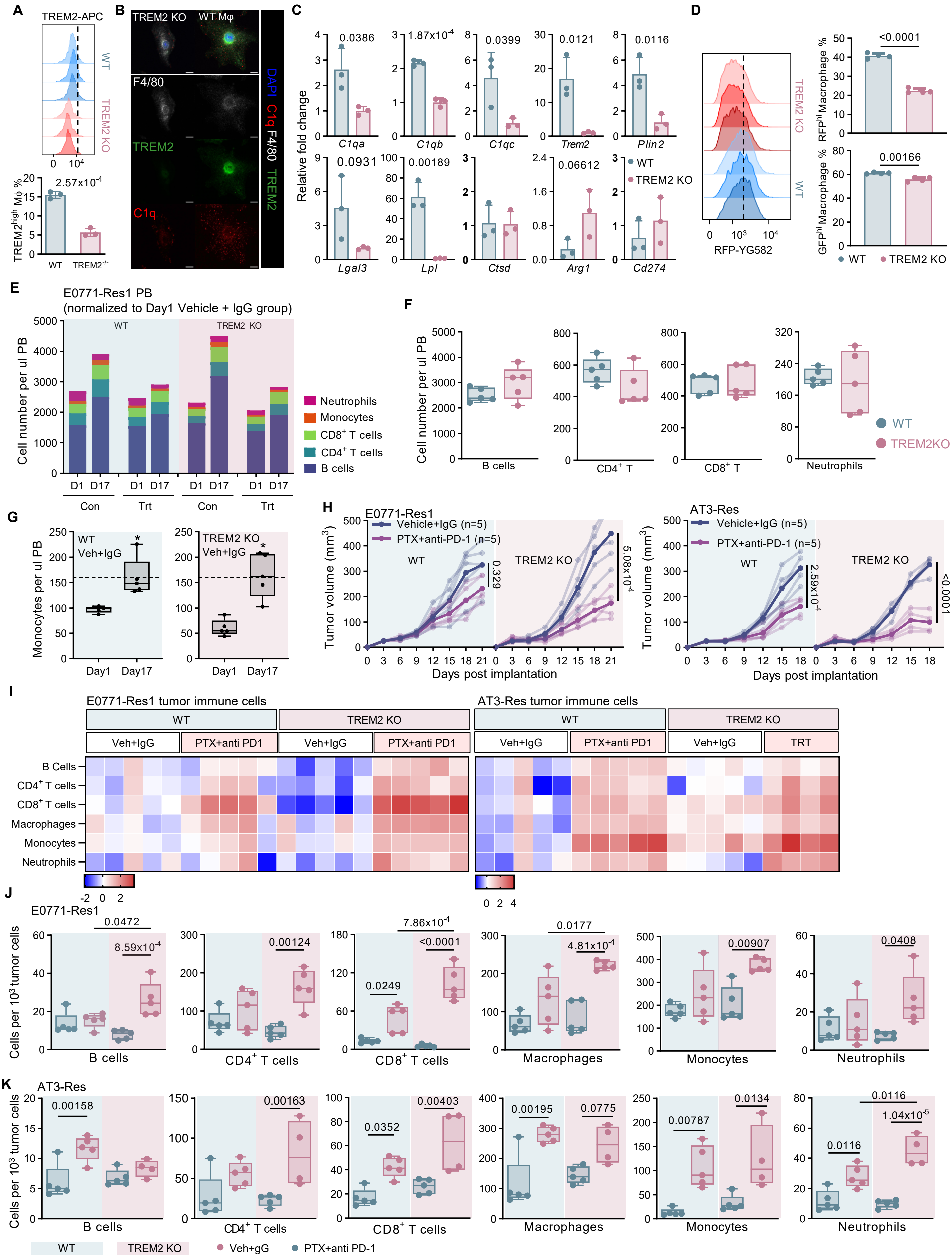
A
TREM2-APC
WT
TREM2 KO
TREM2^high Mϕ %
2.57x10^-4
WT
TREM2^-/-
B
TREM2 KO
WT Mφ
F4/80
TREM2
C1q
DAPI C1q F4/80 TREM2
C
Relative fold change
0.0386
1.87x10^-4
0.0399
0.0121
0.0116
C1qa
C1qb
C1qc
Trem2
Plin2
0.0931
0.00189
0.06612
Lgal3
Lpl
Ctsd
Arg1
Cd274
WT
TREM2 KO
D
RFP-YG582
TREM2 KO
WT
RFP^hi Macrophage %
<0.0001
GFP^hi Macrophage %
0.00166
WT
TREM2 KO
E
E0771-Res1 PB
(normalized to Day1 Vehicle + IgG group)
Cell number per ul PB
WT
TREM2 KO
Neutrophils
Monocytes
CD8+ T cells
CD4+ T cells
B cells
D1 D17 D1 D17 D1 D17 D1 D17
Con
Trt
Con
Trt
F
Cell number per ul PB
B cells
CD4+ T
CD8+ T
Neutrophils
WT
TREM2KO
G
Monocytes per ul PB
WT
Veh+IgG
TREM2 KO
Veh+IgG
Day1
Day17
H
E0771-Res1
Vehicle+IgG (n=5)
PTX+anti-PD-1 (n=5)
WT
TREM2 KO
0.329
5.08x10^-4
Tumor volume (mm^3)
Days post implantation
AT3-Res
Vehicle+IgG (n=5)
PTX+anti-PD-1 (n=5)
WT
TREM2 KO
2.59x10^-4
<0.0001
I
E0771-Res1 tumor immune cells
WT
TREM2 KO
Veh+IgG
PTX+anti PD1
Veh+IgG
PTX+anti PD1
AT3-Res tumor immune cells
WT
TREM2 KO
Veh+IgG
PTX+anti PD1
Veh+IgG
TRT
B Cells
CD4+ T cells
CD8+ T cells
Macrophages
Monocytes
Neutrophils
-2 0 2
0 2 4
J
E0771-Res1
Cells per 10^3 tumor cells
0.0472
8.59x10^-4
B cells
0.00124
CD4+ T cells
7.86x10^-4
<0.0001
0.0249
CD8+ T cells
0.0177
4.81x10^-4
Macrophages
0.00907
Monocytes
0.0408
Neutrophils
K
AT3-Res
Cells per 10^3 tumor cells
0.00158
B cells
0.00163
CD4+ T cells
0.0352
0.00403
CD8+ T cells
0.00195
0.0775
Macrophages
0.00787
0.0134
Monocytes
0.0116
0.0116
1.04x10^-5
Neutrophils
WT
TREM2 KO
Veh+gG
PTX+anti PD-1

Supplemental Figure 6 TREM2$^+$ macrophages mediate resistance against combined therapy, related to Figure 6.

A Representative flow plots (upper panel) and quantitative results (lower panel) of TREM2 signals in BMDMs from wild-type and TREM2 KO mice (n=3 per group).

B Representative immunofluorescence staining of DAPI, C1q, F4/80 and TREM2 in BMDMs collected from wild-type and TREM2 KO mice. Scale bar: 50 μm, 10 μm (zoomed-in).

C Relative expression of murine Cluster 0 macrophage-specific genes in wild-type vs. TREM2 KO macrophages. Each experimental group includes BMDM from three individual mice.

D Representative flow cytometry plots and quantitative results of fluorescence levels in WT vs. TREM2 KO BMDMs co-cultured with RFP-labeled (upper right) or GFP-labeled (lower right) E0771-Res1 cells.

E Flow cytometry analysis of PB immune cells from WT or TREM2 KO mice bearing E0771-Res1 tumors, under control or combined therapy. PB samples were harvested and analyzed on day 1 and 17 post-tumor implantation. Average immune cell number per ul PB were displayed.

F Flow cytometry analysis of peripheral blood immune cells from WT or TREM2 KO mice with E0771-Res1 tumors on day 17 post-tumor implantation. Each experimental group includes five individual mice.

G Flow cytometry analysis of peripheral blood monocytes from WT or TREM2 KO mice with E0771-Res1 tumors on day 1 and 17 post-implantation. Each experimental group includes five individual mice.

H Tumor growth of E0771-Res1 (left) and AT3-Res (right) in wild-type or TREM2 KO mice treated with vehicle or PTX combined with anti-PD-1. Each experimental group includes five individual mice.

I Flow cytometry analysis of immune infiltrates in E0771-Res1 and AT3-Res tumors at the end of experiments. Log2 fold change of major immune cells relative to the vehicle-treated WT group are displayed. Each square represents a specific cell type within an individual tumor.

J and **K** Flow cytometry analysis of major immune cell number per 1000 CD45$^-$ cells in E0771-Res1 (**J**) and AT3-Res (**K**) tumors in wild-type vs. TREM2 KO mice treated with vehicle or PTX combined with anti-PD-1.

(**A**, **C**, **D**, **F**, **G**) Significance was calculated using unpaired two-tailed Student's t test.

(**H**, **J**, **K**) Statistical significance was assessed using one-way ANOVA followed by Tukey's post hoc test.

* $p<0.05$, ** $p < 0.005$, *** $p < 0.001$.

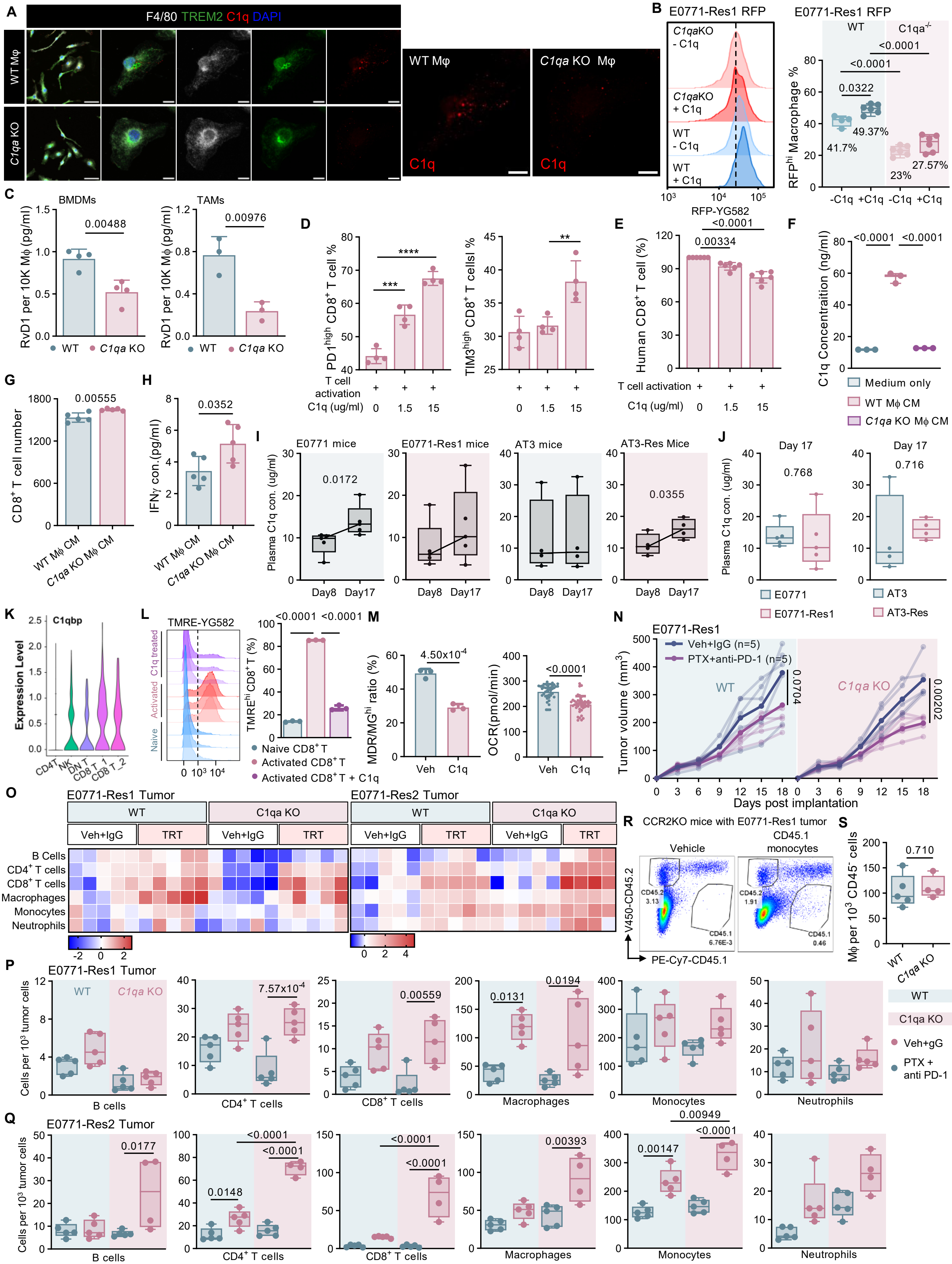

A
F4/80 TREM2 C1q DAPI
WT Mφ
C1qa KO
WT Mφ
C1qa KO Mφ
C1q
B
E0771-Res1 RFP
C1qaKO - C1q
C1qaKO + C1q
WT - C1q
WT + C1q
RFP-YG582
E0771-Res1 RFP
WT
C1qa-/-
RFPhi Macrophage %
<0.0001
<0.0001
0.0322
41.7%
49.37%
23%
27.57%
-C1q +C1q -C1q +C1q
C
BMDMs
RvD1 per 10K Mϕ (pg/ml)
0.00488
WT C1qa KO
TAMs
0.00976
D
PD1high CD8+ T cell %
TIM3high CD8+ T cells %
T cell activation
C1q (ug/ml) 0 1.5 15
E
Human CD8+ T cell (%)
<0.0001
0.00334
T cell activation
C1q (ug/ml) 0 1.5 15
F
C1q Concentration (ng/ml)
<0.0001 <0.0001
Medium only
WT Mϕ CM
C1qa KO Mϕ CM
G
CD8+ T cell number
0.00555
WT Mϕ CM
C1qa KO Mϕ CM
H
IFNγ con.(pg/ml)
0.0352
I
E0771 mice
E0771-Res1 mice
AT3 mice
AT3-Res Mice
Plasma C1q con. (ug/ml)
0.0172
0.0355
Day8 Day17
J
Day 17
0.768
0.716
E0771
E0771-Res1
AT3
AT3-Res
K
C1qbp
Expression Level
CD4T NK DN T CD8 T_1 CD8 T_2
L
TMRE-YG582
C1q treated
Activated
Naive
TMREhi CD8+ T (%)
<0.0001 <0.0001
Naive CD8+ T
Activated CD8+ T
Activated CD8+ T + C1q
M
MDR/MGhi ratio (%)
4.50x10-4
Veh C1q
OCR(pmol/min)
<0.0001
N
E0771-Res1
Veh+IgG (n=5)
PTX+anti-PD-1 (n=5)
WT
C1qa KO
0.0704
0.00202
Tumor volume (mm3)
Days post implantation
O
E0771-Res1 Tumor
E0771-Res2 Tumor
WT
C1qa KO
Veh+IgG
TRT
B Cells
CD4+ T cells
CD8+ T cells
Macrophages
Monocytes
Neutrophils
R
CCR2KO mice with E0771-Res1 tumor
Vehicle
CD45.1 monocytes
V450-CD45.2
PE-Cy7-CD45.1
CD45.2 3.13
CD45.1 6.76E-3
CD45.2 1.91
CD45.1 0.46
S
Mϕ per 103 CD45- cells
0.710
WT
C1qa KO
P
E0771-Res1 Tumor
Cells per 103 tumor cells
WT
C1qa KO
7.57x10-4
0.00559
0.0131
0.0194
B cells
CD4+ T cells
CD8+ T cells
Macrophages
Monocytes
Neutrophils
WT
C1qa KO
Veh+gG
PTX + anti PD-1
Q
E0771-Res2 Tumor
Cells per 103 tumor cells
0.0177
<0.0001
<0.0001
0.0148
<0.0001
<0.0001
0.00393
0.00949
0.00147
<0.0001
B cells
CD4+ T cells
CD8+ T cells
Macrophages
Monocytes
Neutrophils

Supplemental Figure 7 C1q disrupts CD8^{+}T cell metabolism, enabling resistant tumor cells to evade immune surveillance, related to Figure 6.

A Representative immunofluorescence staining of F4/80, TREM2, C1q and DAPI in BMDMs collected from wild-type and *C1qa* KO mice. Scale bar: 50 μm, 10 μm (zoomed-in).

B Representative flow plots (left) and quantitative fluorescence analysis of BMDMs from wild-type and *C1qa* KO mice, following 24 hours of co-culture with PTX pre-treated E0771-Res1 RFP (right). BMDMs were treated with either vehicle or 1 ug/ml C1q protein.

C ELISA quantification of resolvin D1 in BMDMs (left, n=4) and TAMs (right, n=3) from wild-type and *C1qa* KO mice.

D Flow cytometry analysis of the ratio of PD1^{high} and TIM3^{high} to total CD8^{+}T cells following treatment with CD3/CD28 T cell activation beads and indicated concentrations of C1q, n=4.

E Quantification of human CD8^{+}T cells treated with CD3/CD28 T cell activation beads and indicated concentrations of human C1q. CD8^{+} T cells were isolated from the peripheral blood of six healthy female doners.

F ELISA quantification of C1q levels in culture medium collected from blank controls, WT BMDMs, and *C1qa* KO BMDMs. n=3 per group.

G Quantification of total CD8^{+}T cells following treatment with CD3/CD28 beads and culture medium from WT BMDMs and C1qa KO BMDMs. n=5 per group.

H Quantification of IFN-γ production by CD8^{+}T cells activated with CD3/CD28 beads and culture medium from WT BMDMs and C1qa KO BMDMs.

I ELISA quantification of C1q levels in plasma from mice bearing E0771, E0771-Res1, AT3, and AT3-Res tumors. Samples were collected on day8 and day17 post-implantation. E0771 experimental groups include five individual mice; AT3 groups include four individual mice.

J Comparison of C1q levels in plasma from mice bearing E0771, E0771-Res1, AT3, and AT3-Res tumors on day17 post-implantation.

K Violin plots of *C1qbp* expression in T cells and natural killer cells from scRNA sequencing.

L Representative flow plots (left panel) and quantitative analysis (right panel) of TMRE signals in $CD8^+$T cells treated with or without activation beads and 1ug/ml C1q. n=3 per group.

M Flow cytometry analysis of MitoTracker Deep Red (MDR)/ MitoTracker Green (MG) populations and (right) oxygen consumption rate (OCR) in activated T cells treated with vehicle or C1q for 48h.

N Tumor growth of E0771-Res1 in wild-type or C1qa KO mice treated with vehicle or PTX combined with anti-PD-1. Each experimental group includes five individual mice.

O Flow cytometry analysis of the immune infiltrate of E0771-Res1 and E0771-Res2 tumors. The heatmap illustrates log2 fold changes of major immune cells in indicated tumors compared to vehicle-treated wild type controls. Each square represents a specific cell type within an individual tumor.

P and **Q** Flow cytometry analysis of major immune cell number per 1000 $CD45^-$ cells in E0771-Res1 (P) or E0771-Res2 (Q) tumors in wild-type vs. *C1qa* KO mice.

R Flow cytometry analysis of CD45.1 and CD45.2 expression in E0771-Res1 tumor bearing CCR2 KO mice transferred with CD45.1 bone marrow monocytes.

S Flow cytometry analysis of tumor-infiltrating macrophages in CCR2 KO mice bearing E0771-Res1 tumors after transfer of WT monocytes or *C1qa* KO monocytes.

(**B**, **D-F**, **L**, **P**, **Q**) Statistical significance was determined using one-way ANOVA followed by Tukey's test.

(**C**, **G-J**, **M**, **N**, **S**) Significance was calculated using unpaired two-tailed Student's t test.

* $p<0.05$, ** $p < 0.005$, *** $p < 0.001$, **** $p < 0.0001$

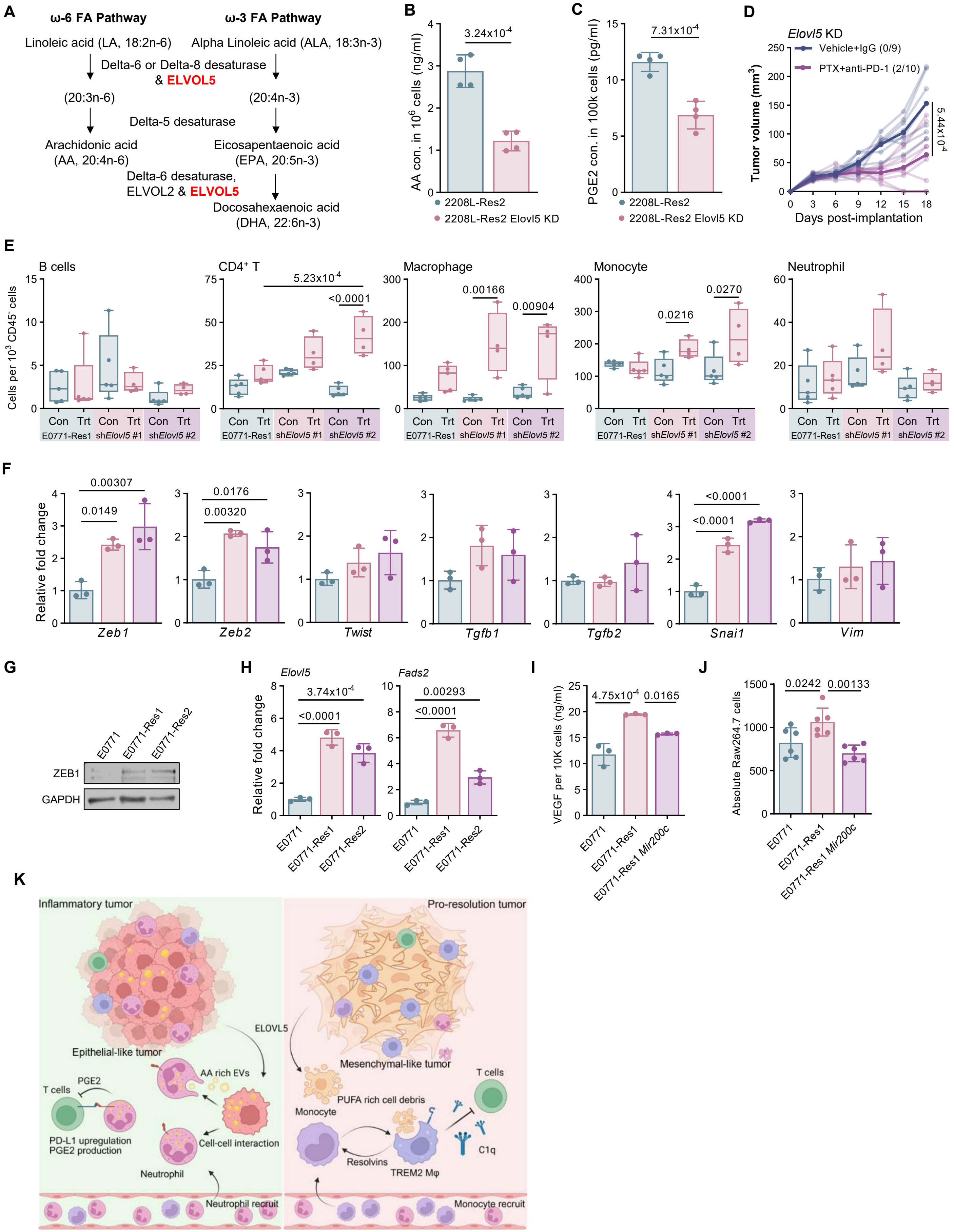
A
ω-6 FA Pathway
ω-3 FA Pathway
Linoleic acid (LA, 18:2n-6)
Alpha Linoleic acid (ALA, 18:3n-3)
Delta-6 or Delta-8 desaturase & ELVOL5
(20:3n-6)
(20:4n-3)
Delta-5 desaturase
Arachidonic acid (AA, 20:4n-6)
Eicosapentaenoic acid (EPA, 20:5n-3)
Delta-6 desaturase, ELVOL2 & ELVOL5
Docosahexaenoic acid (DHA, 22:6n-3)
B
AA con. in 10^6 cells (ng/ml)
3.24x10^-4
2208L-Res2
2208L-Res2 Elovl5 KD
C
PGE2 con. in 100k cells (pg/ml)
7.31x10^-4
2208L-Res2
2208L-Res2 Elovl5 KD
D
Elovl5 KD
Vehicle+IgG (0/9)
PTX+anti-PD-1 (2/10)
Tumor volume (mm^3)
Days post-implantation
5.44x10^-4
E
B cells
CD4+ T
Macrophage
Monocyte
Neutrophil
Cells per 10^3 CD45- cells
5.23x10^-4
<0.0001
0.00166
0.00904
0.0216
0.0270
Con Trt
E0771-Res1
shElovl5 #1
shElovl5 #2
F
Relative fold change
0.00307
0.0149
0.0176
0.00320
<0.0001
<0.0001
Zeb1
Zeb2
Twist
Tgfb1
Tgfb2
Snai1
Vim
G
E0771
E0771-Res1
E0771-Res2
ZEB1
GAPDH
H
Elovl5
Fads2
Relative fold change
3.74x10^-4
<0.0001
0.00293
<0.0001
E0771
E0771-Res1
E0771-Res2
I
VEGF per 10K cells (ng/ml)
4.75x10^-4
0.0165
E0771
E0771-Res1
E0771-Res1 Mir200c
J
Absolute Raw264.7 cells
0.0242
0.00133
E0771
E0771-Res1
E0771-Res1 Mir200c
K
Inflammatory tumor
Pro-resolution tumor
Epithelial-like tumor
Mesenchymal-like tumor
ELOVL5
AA rich EVs
PGE2
T cells
PD-L1 upregulation
PGE2 production
Cell-cell interaction
Neutrophil
Neutrophil recruit
Monocyte
PUFA rich cell debris
T cells
C1q
Resolvins
TREM2 Mφ
Monocyte recruit

Supplemental Figure 8 Elongase 5 protein is required for immunosuppressive microenvironment in resistant tumors, related to Figure 7.

A Synthetic pathways of mammal ω-6 and ω-3 PUFAs.

B ELISA analysis of AA level in 2208L-Res2 and 2208L-Res2 ELOVL5 KD tumor cells.

C ELISA analysis of PGE2 level in 2208L-Res2 and 2208L-Res2 ELOVL5 KD tumor cells.

D Tumor growth of 2208L-Res1 ELOVL5 KD under vehicle or PTX combined with anti-PD-1 antibody, showing both group averages (darker lines) and individual growth curve (lighter lines). Numbers in parentheses indicate cured mice/total mice in each group.

E Flow cytometry analysis of major immune cells infiltrate in E0771-Res1 transduced with control or Elovl5 shRNA tumors under vehicle or combined treatment. Statistical significance was calculated using one-way ANOVA followed by Tukey's test.

F Relative expression of murine mesenchymal marker genes in E0771, E0771-Res1 and E0771-Res2 cells (n=3).

G Immunoblot analysis of ZEB1 protein expression in E0771 and resistant cells. GAPDH serves as the loading control.

H Relative expression of murine *Elovl5* and *Fads2* genes in E0771, E0771-Res1 and E0771-Res2 cells (n=3).

I ELISA quantification of VEGF in cell culture medium of E0771, E0771-Res1 and E0771-Res1 cells with induced microRNA200c expression (n=3).

J Quantification of Raw264.7 cells after trans-well coculture of E0771, E0771-Res1 and E0771-Res1 cells with induced microRNA200c expression (n=6).

K Schematic diagram illustrating the mechanisms of resistance to immuno-chemotherapy in TNBC. Created in https://BioRender.com.

(**B**-**D**) Statistical significance was calculated using Student's t test.

(**E**, **F**, **H**-**J**) Statistical significance was determined using one-way ANOVA followed by Tukey's test.

Supplemental Table 1: Primers used for real-time PCR, related to Methods.

Gene Name	NCBI GeneID	Primer Name	Sequence
Cd274	NM_021893.3	mCD274_F	AGCCTGCTGTCACTTGCTAC
		mCD274_R	AACGCAAGCAGGTCCAGCTC
Plin2	NM_007408	mPlin2_F	AGCTGCTGGTAGACCAGTAC
		mPlin2_R	GAGCAGAGCTTGGTAGACAG
C1qa	NM_007572	mC1qa_F	GTGGCTGAAGATGTCTGCCGAG
		mC1qa_R	TTAAAACCTCGGATACCAGTCCG
C1qb	NM_009777	mC1qb_F	CAACCAGGCACTCCAGGATAA
		mC1qb_R	CCAACTTTGCCTGGAGTCCCAG
C1qc	NM_007574	mC1qc_F	AAGGACGGGCATGATGGACTCC
		mC1qc_R	TTTCCCACGGTGGCCAGGCAT
Trem2	NM_031254	mTREM2_F	CTACCAGTGTCAGAGTCTCCGA
		mTREM2_R	CCTCGAAACTCGATGACTCCTC
Gapdh	NM_008084	mGAPDH_F	AGGTCGGTGTGAACGGATTTG
		mGAPDH_R	TGTAGACCATGTAGTTGAGGTCA
Lpl	NM_008509	mLPL_F	GCGTAGCAGGAAGTCTGACC
		mLPL_R	AGCGTCATCAGGAGAAAGGC
Mrc1	NM_008625	mMRC1_F	GTTCACCTGGAGTGATGGTTC
		mMRC1_R	AGGACATGCCAGGGTCACCT
Lgals3	NM_001145953	mLgals3_F	ACACGAAGCAGGACAATAACTG
		mLgals3_R	GCAGTAGGTGAGCATCGTTGAC
Ctsd	NM_009983	mCTSD_F	TAAGACCACGGAGCCAGTGTCA
		mCTSD_R	CCACAGGTTAGAGGAGCCAGTA
Ctsb	NM_007798	mCTSB_F	AGTCAACGTGGAGGTGTCTGCT
		mCTSB_R	GTAGACTCCACCTGAAACCAGG
Kif1b	NM_207682	mKif1b_F	AGATGACAGCGAGACGACCATG
		mKif1b_R	GCTCATTCACACAGCCGTGGAA
Sele	NM_011345	mSele_F	GGACACCACAAATCCCAGTCTG
		mSele_R	TCGCAGGAGAACTCACAACTGG
Hif1a	NM_010431	mHif1a_F	CCTGCACTGAATCAAGAGGTTGC
		mHif1a_R	CCATCAGAAGGACTTGCTGGCT
Irf1	NM_008390	mIrf1_F	TCCAAGTCCAGCCGAGACACTA
		mIrf1_R	ACTGCTGTGGTCATCAGGTAGG
Nfkb1	NM_008689	mNfkb1_F	GCTGCCAAAGAAGGACACGACA
		mNfkb1_R	GGCAGGCTATTGCTCATCACAG

Supplemental Table 1: Primers used for real-time PCR (Continued)

Gene Name	NCBI GeneID	Primer Name	Sequence
Csf3	NM_009971	mCsf3_F	ATCCCGAAGGCTTCCCTGAGTG
		mCsf3_R	AGGAGACCTTGGTAGAGGCAGA
Il1a	NM_010554	mIl1a_F	ACGGCTGAGTTTCAGTGAGACC
		mIl1a_R	CACTCTGGTAGGTGTAAGGTGC
Lif	NM_008501	mLif_F	TCAACTGGCACAGCTCAATGGC
		mLif_R	GGAAGTCTGTCATGTTAGGCGC
Krt7	NM_033073	mKrt7_F	CGGAGATGAACCGCTCTATCCA
		mKrt7_R	CATGAGCATCCTTGATTGCCAGC
Krt8	NM_031170	mKrt8_F	TGGAAGGACTGACCGACGAGAT
		mKrt8_R	GGCACGAACTTCAGCGATGATG
Krt18	NM_010664	mKrt18_F	AATCAGGGACGCTGAGACCACA
		mKrt18_R	GCTCCATCTGTGCCTTGTATCG
Krt19	NM_008471	mKrt19_F	AATGGCGAGCTGGAGGTGAAGA
		mKrt19_R	CTTGGAGTTGTCAATGGTGGCAC
Emp3	NM_010129	mEmp3_F	GTTCCAACTCTACACCATGCGG
		mEmp3_R	ATCTCCTCGGTGTGGATGGCAT
Tlr3	NM_126166	mTlr3_F	GTCTTCTGCACGAACCTGACAG
		mTlr3_R	TGGAGGTTCTCCAGTTGGACCC
Has2	NM_008216	mHas2_F	CATCTGTGGAGATGGTGAAGGTC
		mHas2_R	AGCCATCCAGTATCTCACGCTG
Cd55	NM_010016	mCd55-F	CCAACTCCTCAGAAACCTTCCAC
		mCd55-R	CCTGTGTTAGGCTCTCCTTTGTC
Edn1	NM_010104	mEdn1-F	CTACTTCTGCCACCTGGACATC
		mEdn1-R	CGCACTGACATCTAACTGCCTG
Itga5	NM_010577	mItga5-F	ACCTGGACCAAGACGGCTACAA
		mIfga5-R	CTGGGAAGGTTTAGTGCTCAGTC
Sphk1	NM_011451	mSphk1-F	GCTTCTGTGAACCACTATGCTGG
		mSphk1-R	ACTGAGCACAGAATAGAGCCGC
Ifnar1	NM_010508	mIfnar1-F	CCAAGGCAAGAGCTATGTCCTG
		mIfnar1-R	CAGTGCGTAGTCTGGACATTTGC
Tlr2	NM_011905	mTlr2-F	ACAGCAAGGTCTTCCTGGTTCC
		mTlr2-R	GCTCCCTTACAGGCTGAGTTCT
Elovl5	NM_134255.3	mElovl5_F	GCGACACTCAACAGCTTCATC
		mElovl5_R	GCAGGTCGTCTGGATGATTG

Supplemental Table 1: Primers used for real-time PCR (Continued)

Gene Name	NCBI GeneID	Primer Name	Sequence
Vim		mVim_F	CGTCCACACGCACCTACAG
		mVim_R	GGGGGATGAGGAATAGAGGCT
Snai1		mSnai1_F	CACACGCTGCCTTGTGTCT
		mSnai1_R	GGTCAGCAAAAGCACGGTT
Twist1		mTwist1_F	GGACAAGCTGAGCAAGATTCA
		mTwist1_R	CGGAGAAGGCGTAGCTGAG
Zeb1		mZeb1_F	GCTGGCAAGACAACGTGAAAG
		mZeb1_R	GCCTCAGGATAAATGACGGC
Zeb2		mZeb2_F	AAACGTGGTGAACTATGACAACG
		mZeb2_R	CTTGCAGAATCTCGCCACTG
Cdh1		mCdh1_F	CAGTTCCGAGGTCTACACCTT
		mCdh1_R	TGAATCGGGAGTCTTCCGAAAA
Fads2	NM_001081664	mFads2_F	GCCCACTCTTTCTGGTTGGAGA
		mFads2_R	AGTAGACAGGCATGAGGAAGGG

Supplemental Table 2: Inflammation resolution marker gene list, related to Methods.

Gene name				
ABHD12	CD93	FCGR3A	MILR1	PYCARD
ABI3	CENPX	FYB1	MMP14	RAB5C
ACP2	CFP	GLIPR1	MRPL23	RAB7B
ACP5	CKB	GNGT2	MRPL42	RAP2B
ADGRE1	CLEC12A	GNS	MRPL51	RASSF4
AHSA1	CLEC4A	GPR65	MRPS33	RGS10
AIF1	CLEC4A	GRN	MRPS36	RNF130
AKR1B1	CLEC4A	GSN	MS4A7	SDC3
APBB1IP	CLTA	HEXA	NCKAP1L	SELENOP
APOE	CNDP2	HEXB	NDUFA12	SERP1
APRT	COA3	HPGDS	NDUFA5	SIRPA
ARRB2	COPS9	IL10RB	NDUFAB1	SLAMF9
ASAH1	COTL1	ITGAM	NDUFC1	SLC11A1
ATP13A2	CTSA	ITGAX	NDUFC2	SNX5
BASP1	CTSB	ITGB5	NDUFS7	TIMP2
BLVRA	CTSD	ITM2B	NFIC	TMED10
BLVRB	CTSH	KCTD12	NFU1	TMEM106A
C1QA	CTSL	LAIR1	NR1H3	TMEM171
C1QB	CTSS	LAMTOR1	P2RY6	TMEM179B
C1QC	CXCL16	LAMTOR4	PF4	TMEM51
C3AR1	CYTH4	LGALS3BP	PGAP2	TMEM86A
C5AR1	DAB2	LGMN	PILRA	TNFAIP8L2
CAMK1	DDRGK1	LIPA	PLD4	TPP1
CCL2	DNAJC19	LMO2	PLTP	TREM2
CCL7	DNASE2	LPL	PLXNB2	TUBB2A
CD180	DTNBP1	LRRC25	POLR2F	TXNIP
CD300C	EHD4	LST1	POU2F2	UAP1L1
CD63	EID1	MAF	PPP1R14B	UNC93B1
CD68	FCER1G	MAN2B1	PRXL2B	VSIR
CD72	FCGR2A	MARCKS	PTPN18	

Supplemental Table 3: The patient IDs and corresponding sample numbers used in Figures 1I and 1K.

Patient ID	Number in Figure 1I, 1K
P1	1
P4	2
Patient1	3
Patient2	4
Patient3	5
Patient5	6
BC3	7
BC5	8
BC8	9
BC_2	10
BC_4	11
BC_5	12
BC_7	13
BC_10	14
BC_11	15
BC_12	16
0106	17
0114	18
0126	19
0135	20
PT039	21
PT081	22
PT089	23
CID3946	24
CID4465	25
CID4495	26
CID4515	27
CID44041	28
CID44971	29

Supplemental Table 4: Antibodies information used in this study.

Antibodies	SOURCE	IDENTIFIER
In Vivo Ready™ Anti-Mouse CD16 / CD32 (Fc Shield)	CYTEK Biosciences	Cat# 40-0161, RRID: AB_2621443
VioletFluor™ 450 Anti-Mouse CD45	CYTEK Biosciences	Cat# 75-0451, RRID: AB_2621947
APC-Cy7 Anti-Mouse CD11b	CYTEK Biosciences	Cat# 25-0112, RRID: AB_2621625
PerCP-Cy5.5 Anti-Mouse Ly-6G	CYTEK Biosciences	Cat# 65-1276, RRID: AB_2621899
PerCP-Cy5.5 Anti-Mouse CD3e	CYTEK Biosciences	Cat# 65-0031, RRID: AB_2621872
APC Anti-Mouse CD4	CYTEK Biosciences	Cat# 20-0041, RRID: AB_2621543
FITC Anti-Mouse CD8a	CYTEK Biosciences	Cat# 35-0081, RRID: AB_2621671
FITC Anti-Mouse F4/80 Antigen	CYTEK Biosciences	Cat# 35-4801, RRID: AB_2621714
APC Anti-Mouse CD366	CYTEK Biosciences	Cat# 20-5870, RRID: AB_2943600
PE Anti-Mouse CD279	CYTEK Biosciences	Cat# 50-9985, RRID: AB_2621814
PE-Cyanine7 Anti-Mouse CD45.1	CYTEK Biosciences	Cat# 60-0453, RRID: AB_2621850
VioletFluor™ 450 Anti-Mouse CD45.2	CYTEK Biosciences	Cat# 75-0454, RRID: AB_2621950
PE-Cyanine7 Anti-Mouse Ly-6C	BioLegend	Cat# 128018, RRID: AB_1732082
PE-Dazzle™ 594 Anti-Mouse CD279	BioLegend	Cat# 135227, RRID: AB_2566005
PE-Cy7 Anti-Mouse CD25	BioLegend	Cat# 102015, RRID: AB_312864
Alexa Fluor® 700 Anti-Mouse CD206	BioLegend	Cat# 141734, RRID: AB_2629637
Brilliant Violet 510™ Anti-Mouse I-A/I-E	BioLegend	Cat# 107635, RRID: AB_2561397
APC Anti-Human/Mouse TREM2	R&D Systems	Cat# FAB17291A, RRID: AB_884527
Lineage Cocktail antibody	BD Bioscience	Cat# 559971, RRID: AB_10053179
Cleaved Caspase-3 (Asp175) Antibody	Cell Signaling	Cat# 9661, RRID: AB_2341188
F4/80 (D2S9R) XP® Rabbit mAb	Cell Signaling	Cat# 70076, RRID: AB_2799771
Perilipin 2 Polyclonal Antibody	Thermo Fisher Scientific	Cat# 15294-1-AP, RRID: AB_2878122
Perilipin-3 Antibody	Novus	Cat# NB110-40764, RRID: AB_715116
F4/80 Antibody	Novus	Cat# NB600-404, RRID: AB_10003219
Human/Mouse MPO Antibody	R&D Systems	Cat# AF3667, RRID: AB_2250866
Mouse TREM2 Antibody	R&D Systems	Cat# AF1729, RRID: AB_354956
Anti-C1q antibody (7H8)	Abcam	Cat# ab11861, RRID: AB_298643
GAPDH Antibody (6C5)	Santa Cruz Biotechnology	Cat# sc-32233, RRID: AB_627679
ELOVL5 Antibody (E-10)	Santa Cruz Biotechnology	Cat# sc-398653, RRID:AB_3662578
C1QBP Antibody (H-9)	Santa Cruz Biotechnology	Cat# sc-271200, RRID: AB_10611471
RFP Antibody	Rockland	Cat# 200-101-379, RRID: AB_2744552
Biotin-SP (long spacer) AffiniPure™ Goat Anti-Rabbit IgG (H+L)	Jackson ImmunoResearch	Cat# 111-065-003, RRID: AB_2337959
Biotin-SP (long spacer) AffiniPure® Donkey Anti-Goat IgG (H+L)	Jackson ImmunoResearch	Cat# 705-065-003, RRID: AB_2340396
AF 488 AffiniPure™ Donkey Anti-Rat	Jackson ImmunoResearch	Cat# 712-545-153, RRID: AB_2340684
AF 488 AffiniPure™ Donkey Anti-Sheep	Jackson ImmunoResearch	Cat# 713-545-147, RRID: AB_2340745
AF647-conjugated Donkey-anti-Rabbit	Jackson ImmunoResearch	Cat# 711-605-152, RRID: AB_2492288
AF555-conjugated Donkey-anti-Rabbit	Thermo Fisher Scientific	Cat# A-31572, RRID: AB_162543
AF555-conjugated Donkey-anti-Sheep	Thermo Fisher Scientific	Cat# A-21436, RRID: AB_2535857
AF555-conjugated Donkey-anti-Goat	Thermo Fisher Scientific	Cat# A-21432, RRID: AB_2535853
AF555-conjugated Donkey-anti-Mouse	Thermo Fisher Scientific	Cat# A-31570, RRID: AB_2536180
InVivoMAb anti-mouse PD-1 (CD279)	Bio X Cell	Cat# BE0146, RRID: AB_10949053
InVivoMAb rat IgG2a isotype control	Bio X Cell	Cat# BE0089, RRID: AB_1107769
InVivoMAb anti-mouse CSF1	Bio X Cell	Cat# BE0204, RRID: AB_2687699
InVivoMAb rat IgG1 isotype control	Bio X Cell	Cat# BE0088, RRID: AB_1107775
