# Supplementary material for: Inflammation- and resolution-programmed myeloid circuits govern therapeutic resistance in epithelial and mesenchymal triple-negative breast cancer
Source: J Clin Invest. 2026 Feb 17;136(8):e198815. doi: 10.1172/JCI198815 (PMC13078875; doi:10.1172/JCI198815)

Full unedited blot/gel for Supplemental Figure 5E

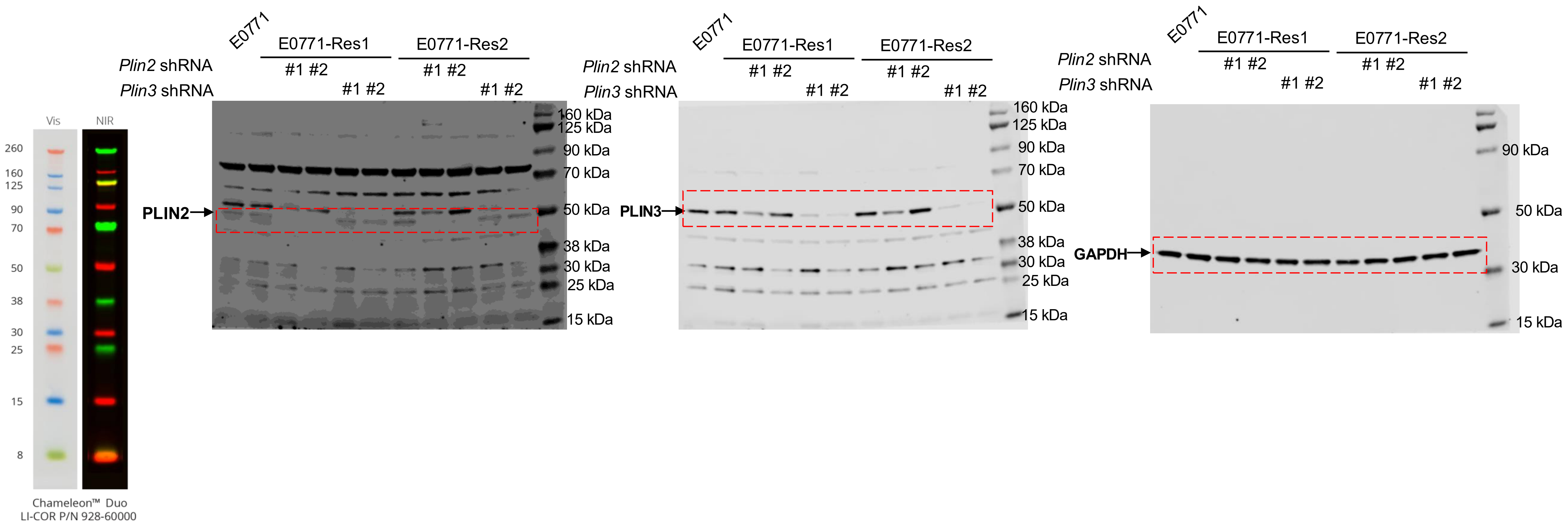

LI-COR Chameleon® Duo Pre-Stained Protein Ladder

Full unedited blot/gel for Figure 6K

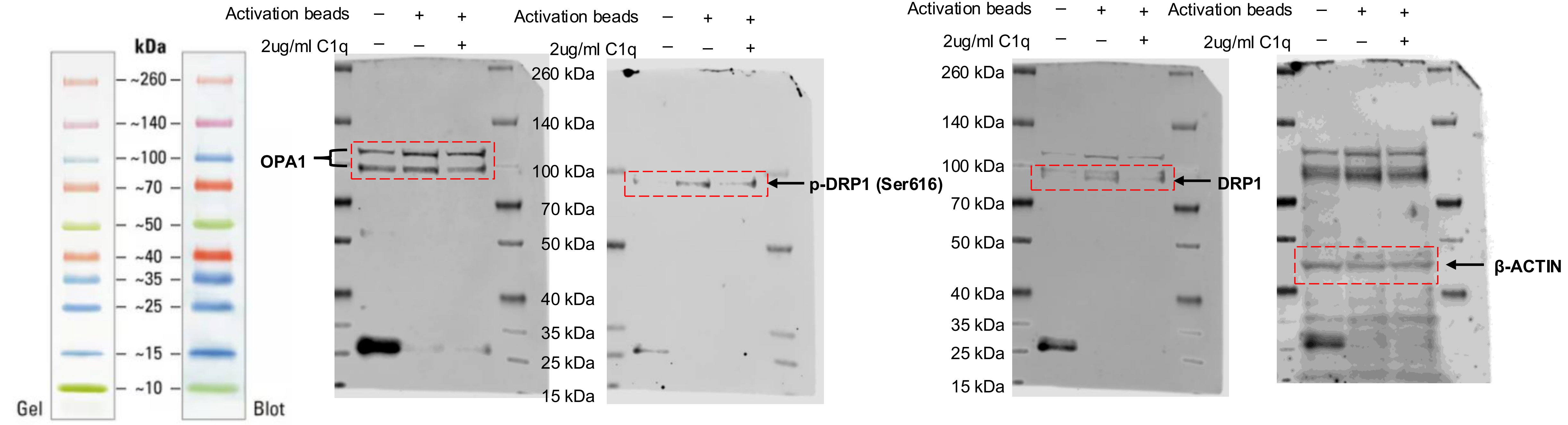

Spectra™ Multicolor Broad Range Protein Ladder

Full unedited blot/gel for Figure 7A

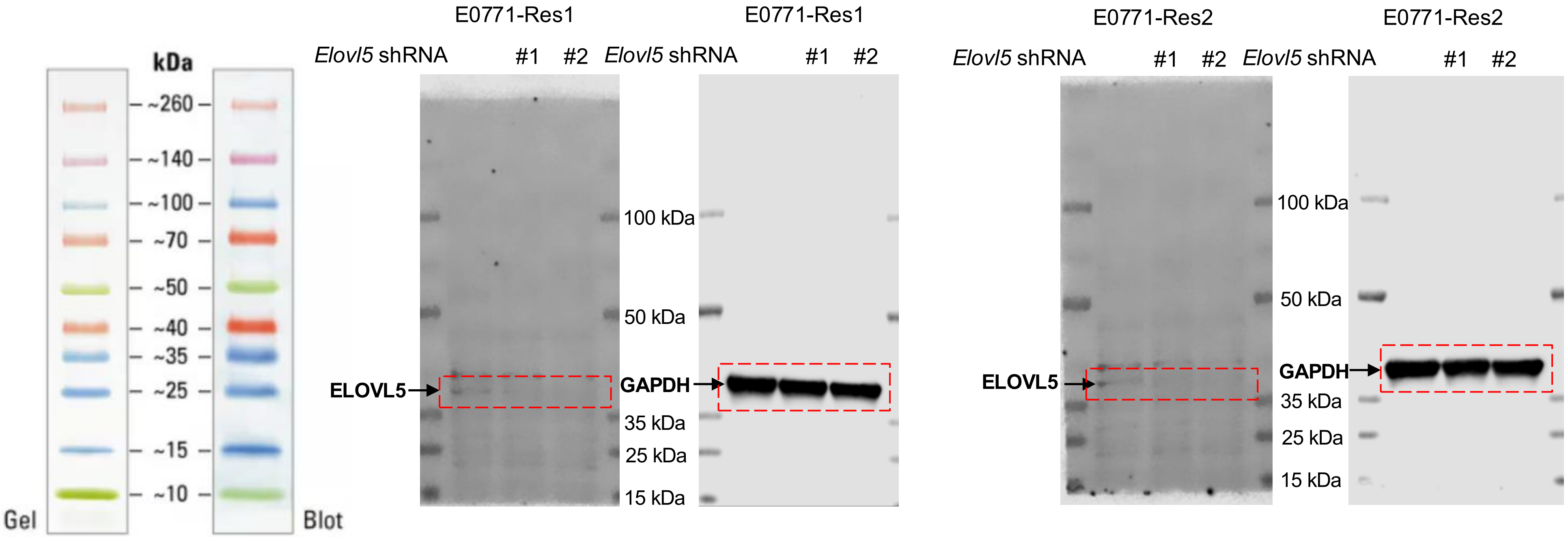

Spectra™ Multicolor Broad Range Protein Ladder

Full unedited blot/gel for Figure 7H

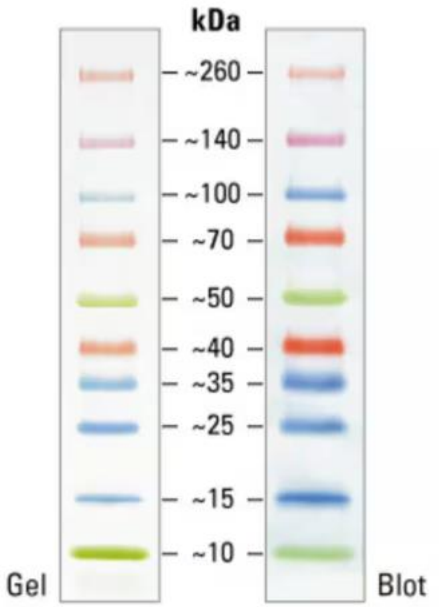

Spectra™ Multicolor Broad Range Protein Ladder

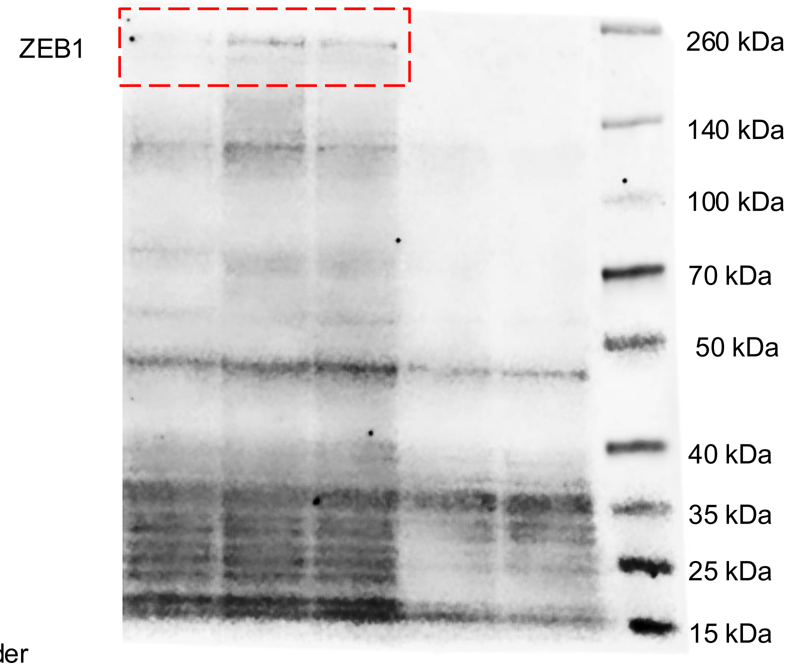

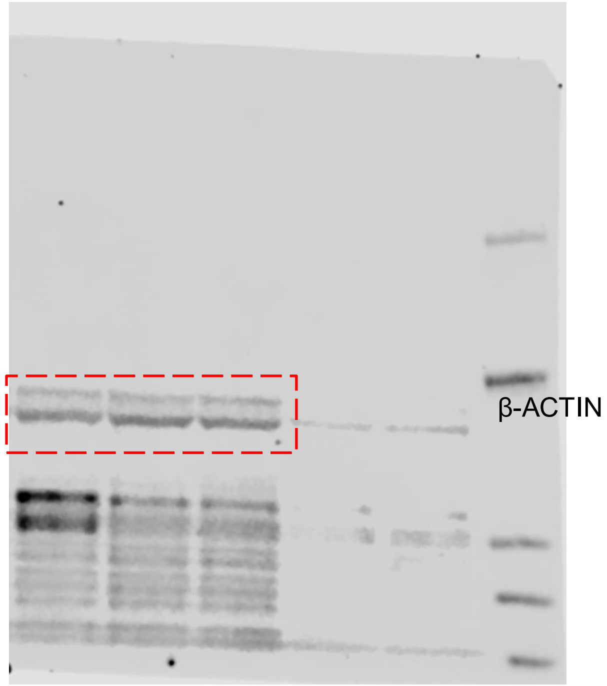

Full unedited blot/gel for Supplemental Figure 8G

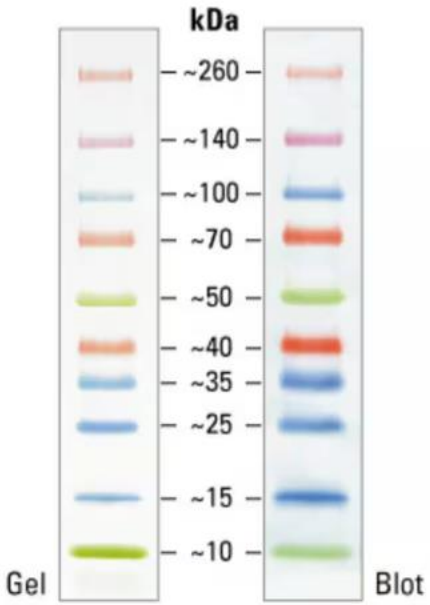

Spectra™ Multicolor Broad Range Protein Ladder

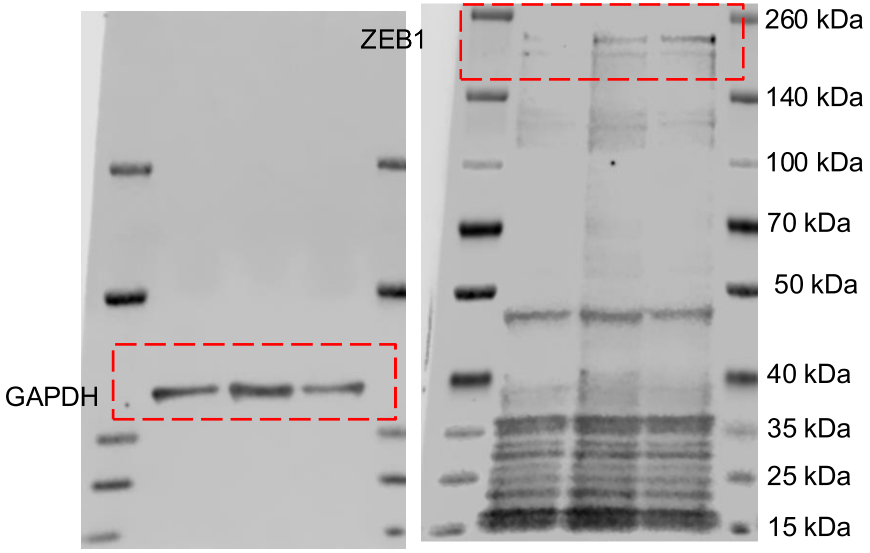

Supplement: Unedited blot and gel images [file jci-136-198815-s129.pdf]
